# Telomere Length, Epigenetic Age Acceleration, and Mortality Risk in US Adult Populations: An Additive Bayesian Network Analysis

**DOI:** 10.1111/acel.70159

**Published:** 2025-07-06

**Authors:** May A. Beydoun, Nicole Noren Hooten, Nigus G. Asefa, Michael F. Georgescu, Minkyo Song, Hind A. Beydoun, Sri Banerjee, Jagdish Khubchandani, Osorio Meirelles, Lenore J. Launer, Michele K. Evans, Alan B. Zonderman

**Affiliations:** ^1^ Laboratory of Epidemiology and Population Sciences National Institute on Aging Baltimore Maryland USA; ^2^ VA National Center on Homelessness Among Veterans U.S. Department of Veterans Affairs Washington District of Columbia USA; ^3^ Department of Management, Policy, and Community Health, School of Public Health University of Texas Health Science Center at Houston Houston Texas USA; ^4^ Public Health Doctoral Programs Walden University Minneapolis Minnesota USA; ^5^ College of Health, Education and Social Transformation New Mexico State University Las Cruces New Mexico USA

**Keywords:** additive Bayesian networks, biological aging, epigenetic clocks, mortality, telomere length

## Abstract

Telomere length and DNA methylation (DNAm) clocks serve as markers of biological aging and have been linked to mortality risk. This study applies additive Bayesian networks (ABNs) to examine associations between DNAm clocks, telomere length, and mortality, with a focus on racial and sex differences in aging. Data from three US cohorts—NHANES (*n* = 2522), HRS (*n* = 1029), and HANDLS (*n* = 92–470)—were analyzed using correlation matrices, Cox models, ABNs, and generalized structural equation models (GSEM) with mortality from the National Death Index. Epigenetic clocks, particularly GrimAgeEAA, HannumAgeEAA, and DunedinPoAM (or DunedinPACE), were stronger mortality predictors than telomere length. ABNs highlighted key relationships, consistently linking age and GrimAgeEAA to mortality in NHANES and HRS. GSEM models derived from ABNs indicated an inverse association between female sex and GrimAgeEAA in NHANES (*β* = −0.500) and HRS (*β* = −0.563), suggesting slower biological aging in women, although GrimAge clock incorporates sex in its definition. GrimAgeEAA strongly predicted mortality (LnHR, *β* ± SE of +0.476 ± 0.0393 in NHANES and +0.511 ± 0.0775 in HRS). Non‐Hispanic Black adults exhibited accelerated aging via DunedinPoAM, partially mediating their higher mortality risk. Hispanic adults in NHANES had unique associations with PhenoAgeEAA (*β* = +0.197), a mortality predictor. DNAm clocks, particularly GrimAgeEAA, outperform telomere length in predicting mortality. Second‐generation epigenetic aging markers offer insights into demographic disparities in aging and mortality, with ABNs revealing complex interrelations among aging biomarkers, sex, race, and mortality risk.

## Graphical abstract

Epigenetic DNA methylation‐based (DNAm) clocks, particularly GrimAge epigenetic age acceleration, outperform telomere length in predicting mortality across three US cohorts. Using Additive Bayesian Networks, we uncovered complex links among biological aging markers, sex, race, and mortality risk, highlighting the utility of DNAm‐based biomarkers in understanding differentials in aging and longevity.

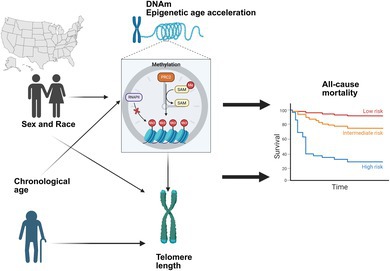

**Abbreviations**
ABN: Additive Bayesian Networks
adaptiveLASSO: adaptive Least Absolute Shrinkage and Selection Operator
cvLASSO: cross‐validation Least Absolute Shrinkage and Selection Operator
DAG: directed acyclic graphs
DIED: Death event binary outcome
DNAm: DNA methylation
DunedinPACE: Dunedin Pace of Aging
DunedinPoAm: Dunedin Pace of Aging DNA methylation
EAA: epigenetic age acceleration
EFTF: Enhanced Face‐to‐Face
GrimAgeEAA: Grim DNA methylation age epigenetic age acceleration
GWAS: Genome‐Wide Association Studies
HANDLS: Healthy Aging in Neighborhoods of Diversity across the Life Span
HannumAgeEAA: Hannum DNA methylation age epigenetic age acceleration
HISP: Hispanic
HorvathAgeEAA: Horvath DNA methylation age epigenetic age acceleration
HR: Hazard Ratio
HRS: Health and Retirement Study
ID: Identification
LASSO: Least Absolute Shrinkage and Selection Operator
Ln: Log
minBICLASSO: minimum Bayesian Information Criterion Least Absolute Shrinkage and Selection Operator
MR: Mendelian Randomization
MRV: Medical Research Vehicles
NDI: National Death Index
NHANES: National Health And Nutrition Surveys
NHB: Non‐Hispanic Black
NHW: Non‐Hispanic White
OTHER: Other race/ethnicities
PhenoAgeEAA: Pheno/Levine DNA methylation age epigenetic age acceleration
PSU: primary sampling units
qPCR: quantitative polymerase chain reaction
RAND: Research and Development
SD: standard deviation
T/S ratio: telomere‐to‐single‐copy gene ratio
TELO_MEAN: Mean telomere length, *z*‐score
US: United States

## 1 Introduction

The average life expectancy in the United States crossed 75 years within the past decade where women continue to outlive men by an average of 5 years (Medina et al. 2020) and widening gaps uncovered in recent years (Yan et al. 2024), coupled with narrowing expectations based on future projections (Medina et al. 2020). Although longer life expectancies are expected (Medina et al. 2020), racial/ethnic differences remain substantial (Beydoun et al. 2016; Luo et al. 2022), differences often mediated by social determinants of health which yield phenotypes of accelerated biological aging (Silva et al. 2023).

The investigation of biological aging and its effects on mortality has resulted in the usage of biomarkers, such as epigenetic clocks and telomere length. Epigenetic clocks, based on DNA methylation (DNAm) data, offer assessments of biological age that frequently correspond more effectively with health outcomes than chronological age (Horvath 2013). These clocks rely on the finding that site‐specific DNAm changes systematically with age. Along with deviations from the predicted age (i.e., epigenetic age acceleration (EAA)), many of them were associated with age‐related disorders, morbidity, and mortality (Levine et al. 2018). Telomere length, a recognized indicator of aging, reflects the gradual reduction of protective chromosomal caps that result from each cell division (Blackburn et al. 2015). Telomere attrition is linked to cellular senescence and oxidative stress, both indicative of increased mortality risk (Blackburn et al. 2015).

Although both epigenetic clocks and telomere length are essential markers of biological aging, their associations with each other and with mortality risk are understudied. Differential factors such as ancestry, socioeconomic level, and exposure to stressors, including environmental pollution or psychosocial distress, can affect these biomarkers and their predictive value for mortality (Fiorito et al. 2017; Needham et al. 2013). Understanding these associations is crucial for recognizing population‐specific aging patterns and risk factors for early mortality.

To address this complexity, we apply additive Bayesian networks (ABNs)—a class of probabilistic graphical models that encode conditional dependencies among variables using directed acyclic graphs (DAGs). ABNs are well suited for exploring multifactorial relationships involving aging, as they integrate prior knowledge with empirical data to uncover network structures reflective of underlying biological and social processes (Delucchi et al. 2022; Kratzer et al. 2023; Scutari and Denis 2021). Their key assumptions include causal sufficiency, acyclicity, and local independence, permitting a semi‐data‐driven approach to identify potential causal pathways and mediators (Delucchi et al. 2022; Kratzer et al. 2023; Scutari and Denis 2021). This approach is especially relevant when modeling high‐dimensional and interrelated data, where conventional regression techniques may falter due to multicollinearity, overfitting, or limited interpretability (Delucchi et al. 2022; Kratzer et al. 2023; Scutari and Denis 2021). In fact, ABNs are especially effective for examining relationships between aging biomarkers and mortality, as it accommodates the hierarchical and interdependent characteristics of biological processes while considering confounding factors and mediating variables, allowing for flexibility in modeling through the leveraging of subject matter knowledge (Delucchi et al. 2022; Kratzer et al. 2023; Scutari and Denis 2021). In contrast to generalized structural equation models (GSEMs), however, subject matter knowledge in ABNs does not have to be absolute. ABNs allow the data to speak for themselves to a large extent, aside from user‐specified constraints such as the hierarchical ordering of variables and the maximum number of parent nodes (i.e., predictors) per variable (Delucchi et al. 2022; Kratzer et al. 2023; Scutari and Denis 2021).

This study examines the correlations among epigenetic clocks, telomere length, and mortality across population subgroups in the US utilizing ABNs. Utilizing this sophisticated statistical framework, we sought to clarify the connections between biological aging indicators and mortality, as well as to determine pathways in sex and racial differences in mortality risk through these biological aging markers.

## 2 Materials and Methods

### 2.1 Databases

#### 2.1.1 National Health and Nutrition Surveys

The National Health and Nutrition Examination Survey (NHANES) comprises a series of cross‐sectional, nationally representative surveys administered by the National Center for Health Statistics (NCHS) from the early 1970s (Beydoun et al. 2016). In 1999, the NHANES transitioned to a continuous series of biennial surveys. Key body measurements were obtained via direct physical examination at a mobile examination facility (See Appendix S1 in Supporting Information for details). Our research included data from the years 1999 to 2002, linked to the death register through 2019. The National Health and Nutrition Examination Survey (NHANES) 1999–2002 data collection followed strict ethical guidelines, including informed consent, confidentiality, risk minimization, and equity, and were approved by the NCHS.

#### 2.1.2 Health and Retirement Study

The Health and Retirement Study (HRS) is a longitudinal panel study that investigates the health, economic, and social factors affecting older Americans (Beydoun et al. 2022). Funded by the National Institute on Aging and the Social Security Administration, the HRS collects data from a representative sample of persons aged 50 and older in the United States (https://hrs.isr.umich.edu/about). The study uses a multistage area probability sample design to ensure it represents the US population over 50 years old. The core data include variables collected from all HRS participants every 2 years, covering various health‐ and retirement‐related domains. Our analysis used the Research and Development (RAND) longitudinal dataset and the Enhanced Face‐to‐Face Interview (EFTF) to gather data on physical, biological, and psychosocial measures and also includes data collected off‐cycle to cover specific factors of interest including biological markers of aging, which are used in the present study, with more details provided in Appendices I, II and III. The study adheres to ethical standards, including informed consent, confidentiality, risk minimization, inclusivity, data use, participant support and longitudinal integrity.

#### 2.1.3 Healthy Aging in Neighborhoods of Diversity Across the Life Span

HANDLS is a longitudinal, interdisciplinary, prospective cohort study including White and African American adults in Baltimore, MD, initiated in 2004. From 2004 to 2009, baseline data (wave 1, w1) were collected by home visits and physical examinations, which included a cognitive test battery conducted on the medical research vehicles (MRV) (Evans et al. 2010). From 2009 to 2013, participants revisited the MRV for a follow‐up in‐person wave (wave 3, w3) and subsequent waves followed a similar protocol (Beydoun, Hossain, et al. 2019; Beydoun et al. 2020). All participants executed written informed consent forms. The HANDLS study protocol was approved by the Institutional Review Board at the National Institutes of Health. In the present study, only data on selected epigenetic clocks and telomere length were used, along with demographic variables, and linkage with all‐cause mortality. The HANDLS sample was mainly used as a validation sample for part of the analysis.

### 2.2 Mortality Linkage

The NHANES links mortality information to participants using the National Death Index (NDI) and a mortality file is provided to be merged with demographics and other variables of interest for each wave of data. The HRS uses NDI linkage, interviews, and public records to track older adults, using a tracker file that can be merged with Core data and the RAND file, among others. The HANDLS study uses NDI and public records to explore mortality differences across various groups. Similarly, this mortality file in HANDLS can be merged with other types of data using individual IDs. These robust linkage strategies allow for comprehensive investigations into survival predictors and mechanisms, especially in the context of socioeconomic and racial/ethnic diversity (See Appendix S1 for more details).

### 2.3 Epigenetic Clocks

Epigenetic clocks are biomarkers of biological aging derived from DNAm patterns at specific CpG sites. These clocks, especially third‐generation clocks, provide insights into the rate of aging and its relationship with health outcomes by estimating epigenetic age and comparing it to chronological age. Major studies in the United States—the HRS, the NHANES, and HANDLS—have utilized these clocks to explore aging‐related gaps in health outcomes. All three studies utilized Illumina MethylEPIC v1.0 BeadChip arrays for DNAm analysis. HRS computed Horvath 1, Hannum, Levine (PhenoAge), GrimAge, and Dunedin Pace of Aging (DunedinPoAm) (Beydoun et al. 2022; Beydoun, Hossain, et al. 2019; Beydoun et al. 2020; Mendy and Mersha 2024). Four clocks were converted into EAA metrics by regressing epigenetic age on chronological age and using the residuals. The Dunedin clock, which already has a measure of the biological aging pace, required no transformation. The NHANES used similar methods to compute EAA for the first four clocks. HANDLS, on the other hand, computed a subset of the clocks (Horvath, Hannum and DunedinPACE) with some modification of those that were provided in NHANES and HRS. DunedinPoAm and DunedinPACE are DNA methylation‐based measures of biological aging rate. DunedinPoAm (2020) estimates aging pace over 12 years (ages, 26–38) (Belsky et al. 2020), while DunedinPACE (2022) extends follow‐up to 20 years (ages, 26–45), improving reliability and predictive validity (Belsky et al. 2022). Both scale 1.0 as 1 year of biological aging per chronological year, but DunedinPACE is preferred for its stronger links to morbidity, mortality, and functional decline (Belsky et al. 2020; Belsky et al. 2022). The calculation of EAA metrics using residuals was consistent across studies for most clocks (See Appendix S2 for details).

### 2.4 Telomere Length

Telomere length is a key biomarker of aging, used in major US population studies like NHANES, HRS, and HANDLS (Wang et al. 2018). These studies use the quantitative polymerase chain reaction (qPCR) method to evaluate the telomere‐to‐single‐copy gene ratio (T/S ratio) as a proxy for relative telomere length. The NHANES and HRS studies used standardized telomere length data to study aging and health outcomes in a population‐representative cohort. The HANDLS study used a different method, assessing relative telomere length from blood samples, to explore differences in aging‐related biomarkers across various groups. Despite differences in sample sources, population characteristics, and analytical processes, all three studies provide valuable insights into the role of telomere length as a biomarker of aging (See Appendix S3 for details). To ensure comparability across datasets, we included only those epigenetic clocks that were available in both NHANES and HRS, and, where possible, also in HANDLS. Clocks that were less commonly used in the literature or based on a limited number of CpG sites were excluded. Notably, although the telomere length‐related clock was available in NHANES, it was not present in HRS or HANDLS and was therefore omitted from our analysis. This selection strategy prioritized clocks with broad validation and cross‐cohort availability.

### 2.5 Covariates

Our analysis included only select demographics as exogenous variables, namely self‐reported age at baseline, sex (0 = Male, 1 = Female), and race/ethnicity. Harmonization of race/ethnicity was done where possible, including between NHANES and HRS, whereby categories of Non‐Hispanic White (NHW), Non‐Hispanic Black (NHB), and Hispanic, as well as “Other ethnicities” were created, resulting in three dummy variables. In HANDLS, only two races/ethnicities were available, namely White and African American. In advanced analyses for NHANES and HRS, White was considered the referent category for analysis of racial differences in mortality risk, biological aging, or both.

### 2.6 Study Samples

Participant flowcharts for NHANES 1999–2002, HRS 2016, and HANDLS 2004–2009 samples are shown in Figure S1. While both NHANES and HRS had epigenetic clock data on individuals aged 50+ years, HANDLS had data at baseline for individuals aged 30–64 years on 363 and 470 participants with data on telomere length and epigenetic clocks, respectively. HANDLS had 92 participants with data on both telomere length and epigenetic clocks, whereas for NHANES, the overlap was consistent between the two types of data. In HRS, of the 4018 participants who had data on epigenetic clock data in 2016, 1029 also had telomere data in 2008, while being aged 58+ in 2016. Figure S1 shows the flow from the initial RAND longitudinal file that included HRS and other earlier data since 1992, to those who were 50+ in the 2008 wave, those who additionally had telomere data during this earlier wave, and finally, those who had epigenetic clocks at the 2016 wave. Follow‐up time also differed across cohorts, with the longest follow‐up times being for NHANES (up to 20 years), followed by HANDLS (up to 18 years), and finally HRS (up to 7 years). Due to the limited sample size in HANDLS, analyses in this sample were mainly used to validate part of the analyses conducted in NHANES and HRS (correlation matrix, Kaplan–Meier curves/log‐rank tests, LASSO linear, Cox models for each predictor on the largest available HANDLS sample, adjusting for exogenous variables age, sex, and race).

### 2.7 Statistical Methods

All analyses were carried out using Stata release 18.0 (StataCorp 2023), while visualizations were partly produced using R version 4.4.1 (R Core Team 2024). As a first step, descriptive analyses summarized the distributions of key variables of interest, including means, medians, standard deviations, interquartile ranges as well as frequency distributions for categorical data. Visualizations for each variable included histograms which were used to identify outliers and standard processes were used to remove outliers across all continuous variables of interest. Given that three cohorts of data were used, descriptive statistics also included comparisons of baseline characteristics and key variables across these cohorts, using linear models for continuous variables and multinomial logit models for categorical variables, while accounting for sampling design complexity to obtain population estimates (sampling weights, primary sampling units (PSUs) and strata for HRS and NHANES, and sampling weights for HANDLS). For HANDLS, comparisons were made with the other cohorts using the largest available sample with data on either epigenetic clocks or telomere length (*N* = 741 for demographics and mortality rate, *N* = 470 for epigenetic clocks, *N* = 363 for telomere length).

As a second step, Kaplan–Meier survival curves were conducted for all three cohorts, accounting for sampling weights, to estimate the probability of survival over time, accounting for censored observations, and more importantly by comparing those survival experiences across tertiles of biological aging metrics (epigenetic DNAm age acceleration and mean telomere length), and assessing whether differences in survival times were statistically significant using log‐rank tests. This part of the analysis was adjusted for sampling design complexity by including sampling weights, and the largest available sample with epigenetic clocks or telomere length was used for the HANDLS cohort.

Third, the interrelationships of various biological aging metrics were quantified using Pearson's correlations across the three cohorts of data and visualized using correlation heat maps. No sampling weight adjustment was made in this part of the analysis and the smaller sample with both epigenetic clocks and telomere length data was used for HANDLS (*n* = 92).

Fourth, multivariable‐adjusted Cox proportional hazards models were conducted after testing the proportionality of the hazards through Schoenfeld residuals. These models were adjusted for age, sex, and race/ethnicity, and the main exposures were each of the six biological aging metrics (i.e., epigenetic DNAm age acceleration and telomere length), with the outcome being time to all‐cause mortality, an analysis also adjusted for sampling weights. Two related sensitivity analyses were conducted to assess the robustness of the associations between biological aging markers and mortality. As a first step, the DunedinPoAm measure was regressed on chronological age, and the resulting residuals were analyzed in Cox proportional hazards models to evaluate age‐independent effects. As a second step, Harrell's *C*‐statistics were estimated from Cox models including each aging biomarker along with covariates (age, sex, race/ethnicity) to assess model discrimination. The concordance statistic (C) reflects the model's ability to correctly rank survival times and is interpreted similarly to the area under the ROC curve (AUC). These sensitivity analyses were mainly implemented in HRS and NHANES cohorts.

Fifth, Least Absolute Shrinkage and Selection Operator (LASSO) linear regression was employed to identify the most predictive clock measures for telomere length as the outcome of interest (Appendix S4). By penalizing the inclusion of less relevant variables, LASSO decreases model complexity and avoids overfitting. This analysis forces the inclusion of exogenous variables, namely age, sex, and race/ethnicity to ensure robust selection while accounting for known potential confounders. The process is applied to a random half sample using cross‐validation (cvLASSO), minimum Bayesian information criterion (minBICLASSO), and adaptive LASSO (adaptiveLASSO) algorithms. The simplest (i.e., with the least number of additional parameters) of the three models is then applied to the full sample, and model fit is compared across the two half samples. This part of the analysis is applied to all three cohorts, without sampling weight adjustment.

Sixth, ABNs were employed to model the complex interplay between predictors, mediators, and outcomes (Delucchi et al. 2022; Kratzer et al. 2023; Scutari and Denis 2021) and https://r‐bayesian‐networks.org/. In the context of discrete time hazards, ABNs allow for the estimation of probabilistic relationships among variables while accounting for time‐dependent survival risks after modifying data into a person‐period format and the inclusion of a 2‐year period of follow‐up binary dummy covariates as is often done in discrete time hazards models (Appendices V and VI). All variables included in ABN were therefore either Gaussian or binomial and continuous variables were discretized using percentiles which were represented by their median values. Model fit was assessed for 1 through 3 parents/child and 2 parents/child was selected if there was a leveling off of model fit between 2 and 3 parents/child. This part of the analysis was carried out only on NHANES and HRS cohorts. This part of the analysis was not adjusted for sampling weights or sampling design complexity.

Finally, using a Weibull regression modeling framework for the mortality outcome, generalized structural equations were carried out to replicate the final selected ABNs and estimate standard errors for each of the relationships that were uncovered in the final DAGs (Appendix S7). This model was carried out specifically to estimate the relationships among biological aging metrics, between biological aging metrics and mortality, and the pathways between age, sex, and racial contrasts and all‐cause mortality through biological aging metrics. This part of the analysis was adjusted for sampling design complexity (sampling weights, PSUs, and strata) and compared with a model that assumed simple random samples. A type I error of less than 0.05 was considered statistically significant.

## 3 Results

Across the three cohorts—NHANES, HRS, and HANDLS—we observed important differences in sociodemographic factors, mortality, and biological aging markers (Table 1). Participants in the HRS cohort were generally older than those in NHANES and HANDLS and included a higher proportion of women (significantly more than NHANES). HRS also exhibited the highest mortality rate among the three cohorts. In contrast, HANDLS had the largest proportion of non‐Hispanic Black (NHB) adults, while NHANES included the highest proportion of Hispanic participants. Telomere length z‐scores revealed that NHANES participants had the shortest telomeres on average, though direct comparisons across cohorts were complicated by differences in measurement methodology—particularly in HANDLS, which required z‐score standardization for comparability. Notably, the four epigenetic age acceleration (EAA) metrics and the DunedinPoAm (or DunedinPACE in HANDLS) measure did not differ significantly across cohorts in terms of their mean values.

**TABLE 1:** Study characteristics and mortality risk across three cohorts (NHANES, HRS, and HANDLS).

	NHANES 1999–2002	HRS 2008 and 2016	HANDLS 2004–2009
	Mean ± SE	Mean ± SE or %	Mean ± SE or %
Demographics	(*n* = 2522)	(*n* = 1029)	(*n* = 741)
Age	64.0 ± 0.3	73.1 ± 0.47***	45.9 ± 0.7***
Sex, % female	54.4	59.0*	51.1
Race/ethnicity			
Non‐Hispanic White	78.5	82.5	37.7
Non‐Hispanic Black	8.7	7.7	62.3***
Hispanic	9.4	7.3*	0.0***
Other	3.3	2.5***	0.0***
Epigenetic age acceleration metrics	(*N* = 2522)	(*N* = 1029)	(*N* = 470)
HorvathAgeEAA	0.22 ± 0.19	0.12 ± 0.25	0.10 ± 0.36
HannumAgeEAA	−0.18 ± 0.16	0.16 ± 0.20	−0.26 ± 0.36
PhenoAgeEAA	−0.20 ± 0.20	−0.14 ± 0.27	__
GrimAgeEAA	−0.32 ± 0.17	−0.44 ± 0.19	__
DunedinPoAm (or DunedinPACE)	1.10 ± 0.004	1.07 ± 0.004	1.05 ± 0.01
Telomere length metrics	(*N* = 2522)	(*N* = 1029)	(*N* = 363)
Telomere length	0.940 ± 0.017	1.34 ± 0.01	5.66 ± 0.07
Telomere length, *z*‐score	−0.336 ± 0.064	+0.015 ± 0.037***	+0.025 ± 0.091***
	(*N* = 2522)	(*N* = 1029)	(*N* = 741)
Mortality rate, per 1000 Person‐years, with 95% CI	32.2 (30.0–34.8)	37.7 (32.7–42.5)	9.3 (7.2–12.0)
Hazard Ratio, with 95% CI	1.00	1.69 (1.39; 2.04)***	0.29 (0.22;0.38)***

*Note:* Differences in means and proportions across cohorts were tested by taking NHANES as the referent category through bivariate linear and multinomial logistic regression models with “COHORT” as the only predictor entered as two dummy variables: COHORT2 (HRS vs. NHANES) and COHORT3(HANDLS vs. NHANES). Telomeres were measured in 2008 in HRS while epigenetic clocks and baseline age, as well as all other covariates, were measured in 2016. Hazard ratios were estimated using Cox proportional hazards models with COHORT as the only predictor (also as two dummy variables). HANDLS computed DunedinPACE instead of DunedinPoAm.

Abbreviations: CI, confidence Interval; DunedinPACE, Dunedin Pace of Aging, used in HANDLS; DunedinPoAm, Dunedin Pace of Aging DNA methylation clock; GrimAgeEAA, Grim DNA methylation Epigenetic Age Acceleration; HANDLS, Healthy Aging in Neighborhoods of Diversity across the Life Span; HannumAgeEAA, Hannum DNA methylation Age; HorvathAgeEAA, Horvath DNA methyalation Age Epigenetic Age Acceleration; HRS, Health and Retirement Study; *n*, unweighted sample; NHANES, National Health and Nutrition Examination Surveys; PhenoAgeEAA, Pheno DNA methylation Age Epigenetic Age Acceleration; SE, Standard Error.

*p < 0.05; **p < 0.010; ***p < 0.001 for null hypothesis of no difference in means or proportions between HRS or HANDLS and the referent cohort NHANES.

**FIGURE 1:**
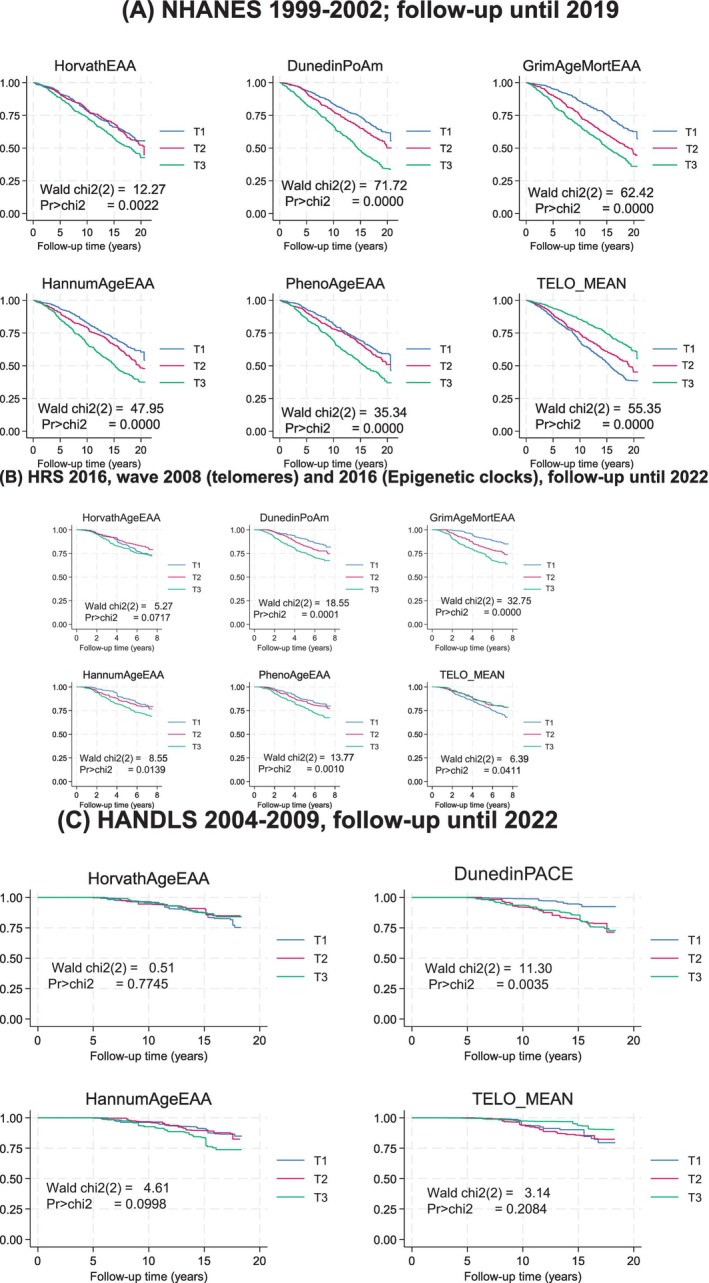
Kaplan–Meier survival curves across tertiles of markers of biological aging and three cohorts: NHANES 1999–2019, HRS 2016–2022, and HANDLS 2004–2022. Kaplan–Meier survival curves were conducted in all three cohorts with time on study considered as the time variable to event (all‐cause death) or censoring by end of follow‐up. Maximum follow‐up time ranged from ~8 years for HRS (starting from baseline age in 2016) to 20 years for NHANES. Median values for tertiles (T1/T2/T3) were 0.93 to 1.02/1.06 to 1.10/1.16 to 1.21 for DunedinPoAm across cohorts; −4.38 to −4.37/−0.96 to −0.83/4.48 to 4.74 to for GrimAgeEAA; –5.4 to −4.52/−0.25 to +0.63/4.46 to 5.16 for HannumAgeEAA; −5.27 to −4.31 /−0.12 to 0.15/4.40 to 5.53 for HorvathAgeEAA; −6.11 to −6.07/–0.32 to +0.04/5.85 to 6.12 for PhenoAgeEAA. TELO_MEAN tertile medians were + 0.72 to 1.06/0.89 to 1.29/1.13 to 1.61 for NHANES and HRS and 5.0/5.66/6.3 for HANDLS. Sampling weights were accounted for in this analysis. Unweighted sample sizes were *n* = 2522 for NHANES, *n* = 1029 for HRS and *n* = 363 (telomere length) 470 (epigenetic clocks) for HANDLS. HANDLS computed DunedinPACE instead of DunedinPoAm. Chi2, Chi‐square; DunedinPACE, Dunedin Pace of Aging; DunedinPoAm, Dunedin Pace of Aging DNA methylation clock; GrimAgeEAA, Grim DNA methylation Epigenetic Age Acceleration; HANDLS, Healthy Aging in Neighborhoods of Diversity across the Life Span; HannumAgeEAA, Hannum DNA methylation Age, Epigenetic Age Acceleration; HorvathAgeEAA, Horvath DNA methylation Age, Epigenetic Age Acceleration; HRS, Health and Retirement Study; NHANES, National Health and Nutrition Examination Surveys; PhenoAgeEAA, Pheno DNA methylation Age Epigenetic Age Acceleration; T1, First tertile; T2, Second tertile; T3, Third tertile; TELO_MEAN, Mean telomere length. Panels A, B and C are for NHANES, HRS and HANDLS cohorts, respectively.

Importantly, Kaplan–Meier survival curves (Figure 1) demonstrated that several biological aging markers were associated with mortality risk, though the strength and direction of these associations varied by cohort. In NHANES, shorter telomeres were strongly predictive of increased mortality, whereas in HRS, this association was weaker. For epigenetic clocks, HorvathAgeEAA showed a marginal relationship with mortality in HRS but was not significant in HANDLS. Interestingly, in HANDLS, only the DunedinPACE metric was significantly associated with mortality; no such associations were observed for telomere length or other EAA measures.

**FIGURE 2:**
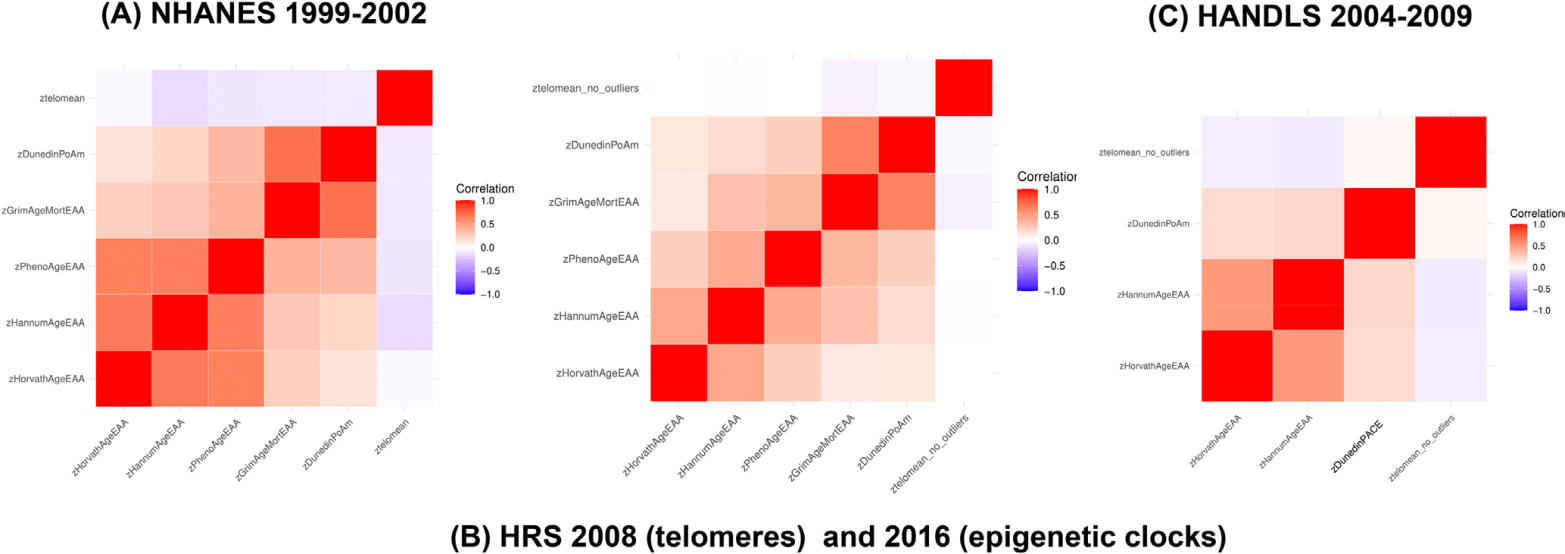
Pearson's correlation matrix between epigenetic clock metrics and telomere length: NHANES 1999–2002, HRS 2008 and 2016, and HANDLS 2004–2009. Sampling weights were not accounted for in this analysis. Unweighted sample sizes were *n* = 2522 for NHANES, *n* = 1029 for HRS and *n* = 92 for HANDLS. HANDLS computed DunedinPACE instead of DunedinPoAm. DunedinPACE, Dunedin Pace of Aging; DunedinPoAm, Dunedin Pace of Aging DNA methylation clock; GrimAgeEAA, Grim DNA methylation Epigenetic Age Acceleration; HANDLS, Healthy Aging in Neighborhoods of Diversity across the Life Span; HannumAgeEAA, Hannum DNA methylation Age, Epigenetic Age Acceleration; HorvathAgeEAA, Horvath DNA methylation Age, Epigenetic Age Acceleration; HRS, Health and Retirement Study; NHANES, National Health and Nutrition Examination Surveys; PhenoAgeEAA, Pheno DNA methylation Age Epigenetic Age Acceleration; TELO_MEAN, Mean telomere length; *z*, standardized *z*‐score. Panels A, B and C are for NHANES, HRS and HANDLS cohorts, respectively.

Furthermore, Figure 2 presents the correlations among the biological aging markers. Strong correlations were seen between HorvathAgeEAA and HannumAgeEAA (*r* > 0.80) and between GrimAgeEAA and DunedinPoAm, especially in NHANES and HRS. Correlations between telomere length and the clocks were notably weaker. The only exception was a weak inverse correlation between telomere length and the Hannum clock in NHANES (*r* ≈ −0.20), suggesting potential divergence in the biological processes captured by telomere and epigenetic aging metrics.

These patterns were echoed in our LASSO regression analyses (Figure S2). In NHANES, HannumAgeEAA emerged as the strongest independent predictor of telomere length (after adjusting for age, sex, race/ethnicity, and other clocks), while age was the only variable consistently and inversely associated with telomere length across all three cohorts. In the HANDLS subsample (*n* = 92), telomere length was not associated with any of the available clocks after adjusting for demographics.

**FIGURE 3:**
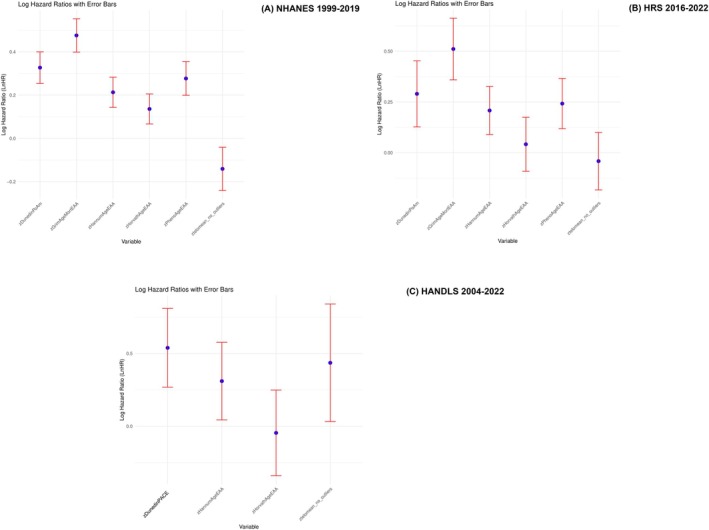
Association of each biological aging metric with mortality risk adjusting for key exogenous variables: Cox proportional hazards models. Models are adjusted for age, sex, and race/ethnicity within each cohort. Values are Ln(hazard ratios) with 95% CI for each biological aging metric. Note that GrimAgeEAA and PhenoAgeEAA were only measured in NHANES and HRS. Sampling weights were accounted for in this analysis. Unweighted sample sizes were *n* = 2522 for NHANES, *n* = 1029 for HRS, and *n* = 363 (telomere length) 470 (epigenetic clocks) for HANDLS. HANDLS computed DunedinPACE instead of DunedinPoAm. Dunedin PACE, Dunedin Pace of Aging; DunedinPoAm, Dunedin Pace of Aging DNA methylation clock; GrimAgeEAA, Grim DNA methylation Epigenetic Age Acceleration; HANDLS, Healthy Aging in Neighborhoods of Diversity across the Life Span; HannumAgeEAA, Hannum DNA methylation Age, Epigenetic Age Acceleration; HorvathAgeEAA, Horvath DNA methylation Age, Epigenetic Age Acceleration; HRS, Health and Retirement Study; NHANES, National Health and Nutrition Examination Surveys; PhenoAgeEAA, Pheno DNA methylation Age Epigenetic Age Acceleration; TELO_MEAN, Mean telomere length; *z*, standardized *z*‐score. Panels A, B and C are for NHANES, HRS and HANDLS cohorts, respectively.

Cox proportional hazards models (Figure 3) further emphasized the predictive utility of epigenetic clocks over telomere length for mortality risk. GrimAgeEAA and PhenoAgeEAA were consistently and significantly associated with increased mortality in both NHANES and HRS. For example, GrimAgeEAA had hazard ratios translating to *β* ± SE of +0.476 ± 0.0393 in NHANES and +0.511 ± 0.0775 in HRS. HannumAgeEAA and DunedinPoAm were also significantly associated with mortality risk across all three cohorts. In contrast, telomere length showed an inverse association with mortality in NHANES (shorter telomeres → higher risk), but a counterintuitive positive association in HANDLS.

In sensitivity analysis #2, Harrell's C‐statistics were computed to evaluate the discriminatory performance of epigenetic aging biomarkers for mortality. Among HRS participants, GrimAgeEAA exhibited the highest concordance (*C* = 0.7641), followed by DunedinPoAm (*C* = 0.7476), PhenoAgeEAA (*C* = 0.7451), and HannumAgeEAA (*C* = 0.7378). HorvathAgeEAA and telomere length showed lower *C*‐statistics (*C* = 0.7278 and *C* = 0.7281, respectively). In NHANES, GrimAgeEAA also had the highest concordance (*C* = 0.7628), slightly outperforming DunedinPoAm (*C* = 0.7544), with PhenoAgeEAA (*C* = 0.7501) and HannumAgeEAA (*C* = 0.7463) following closely. Adjustment of DunedinPoAm for age (residualized version, sensitivity analysis #1) did not affect its *C*‐statistic in either cohort. These findings confirm that GrimAgeEAA and DunedinPoAm consistently provided the strongest mortality discrimination, supporting their utility in aging research across diverse US populations. These findings are provided as raw Output in: https://github.com/baydounm/HRS_NHANES_HANDLS_TLEPIGENMORT/tree/main.

**FIGURE 4:**
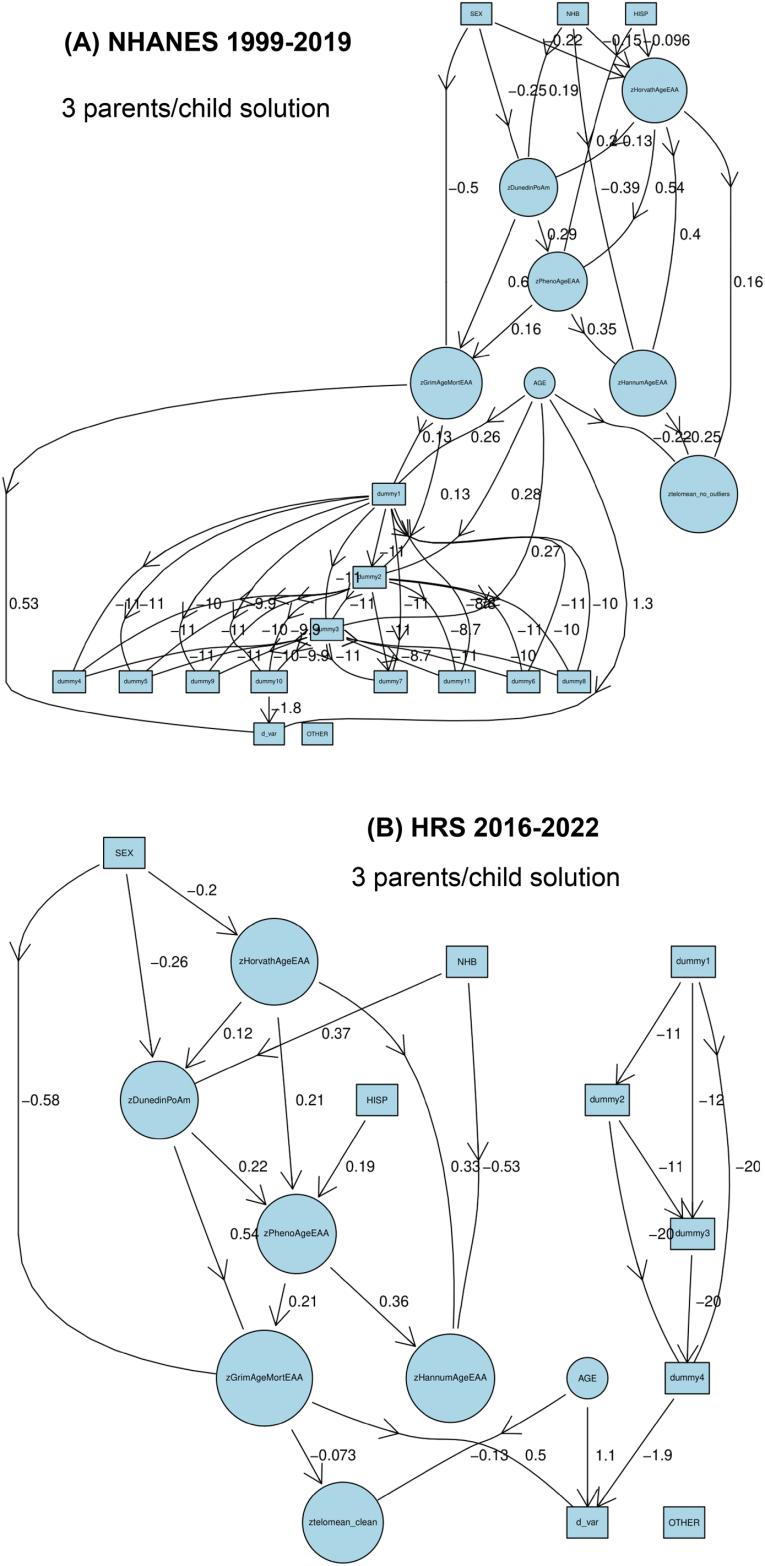
Additive Bayesian network solutions for three parents/child for associations among biological aging metrics, demographics and mortality risk (discrete time hazards). Details for R code used for this analysis described in Appendix S6 and provided on github. This code provides a comprehensive pipeline for conducting ABN analysis, including installation, data preprocessing, constraint specification, model fitting, and iterative optimization. It involves installing R versions 4.4 or higher, data preparation, data wrangling, defining variable groups, setting constraints, optimizing across parent limits, building the additive Bayesian network, and generating visual representations. The optimal number of parents of a child is determined based on leveling off the log marginal likelihood and desired complexity between key variables. Unweighted sample sizes were *n* = 2522 for NHANES and *n* = 1029 for HRS. DunedinPoAm, Dunedin Pace of Aging DNA methylation clock; GrimAgeEAA, Grim DNA methylation Epigenetic Age Acceleration; HannumAgeEAA, Hannum DNA methylation Age, Epigenetic Age Acceleration; HorvathAgeEAA, Horvath DNA methylation Age, Epigenetic Age Acceleration; HRS, Health and Retirement Study; NHANES, National Health and Nutrition Examination Surveys; PhenoAgeEAA, Pheno DNA methylation Age Epigenetic Age Acceleration; TELO_MEAN, Mean telomere length; *z*, standardized *z*‐score. Panels A and B are for NHANES and HRS cohorts, respectively.

Using additive Bayesian network (ABN) modeling (Figure 4; Figures S3 and S4), we subsequently explored how biological aging metrics, demographics, and mortality interconnected. In both NHANES and HRS, allowing up to three parent nodes per outcome improved model fit, suggesting nuanced interdependencies. Age and GrimAgeEAA consistently emerged as dominant predictors of mortality risk (d_var). Female sex was linked to lower GrimAgeEAA and thus lower mortality, while NHB participants tended to have slower aging as measured by DunedinPoAm. Interestingly, in HRS, NHB participants had shorter telomeres, and Hispanic participants had lower HannumEAA values. However, these findings did not uniformly translate into mortality risk differences across clocks or cohorts. It is worth noting that biological sex is incorporated in the definition of GrimAge.

Based on Table 2, Weibull models within the GSEM framework, provided complementary insight and replicated the ABN findings. Telomere length remained inversely associated with age (*β* = −0.220 in NHANES; *β* = −0.140 in HRS), although its link to mortality was less pronounced than for the epigenetic clocks. GrimAgeEAA stood out as the most robust mortality predictor, followed by PhenoAgeEAA, whose effects were mediated through HannumAgeEAA and DunedinPoAm. The latter two metrics also played key intermediary roles in linking demographic characteristics to aging and mortality. We also identified consistent sex and racial/ethnic patterns. Female participants showed significantly lower GrimAgeEAA (*β* = −0.500 in NHANES; *β* = −0.563 in HRS) and DunedinPoAm (*β* = −0.270 in NHANES; *β* = −0.258 in HRS), pointing to slower biological aging. Non‐Hispanic Black individuals had faster aging by DunedinPoAm (*β* = +0.192 in NHANES; *β* = +0.347 in HRS), but lower HannumAgeEAA (*β* = −0.358 in NHANES; *β* = −0.498 in HRS), suggesting multidimensional and sometimes offsetting effects. In NHANES, Hispanic adults exhibited elevated PhenoAgeEAA (*β* = +0.197), a known mortality predictor, underscoring the potential for race/ethnicity‐specific aging pathways.

**TABLE 2:** Generalized structural equations models in NHANES and HRS sample based on the three‐parents/child limit Additive Bayesian Network Model solution for each cohort^a^.

	Model 1^b^		Model 2^c^	
	*β* ± SE	*p* ^d^	*β* ± SE	*p* ^d^
NHANES 1999–2019 (*n* = 2522)				
AGE ➔ TELO_MEAN	−0.220 ± 0.016	< 0.001	−0.207 ± 0.022	< 0.001
AGE ➔ DIED	+0.978 ± 0.031	< 0.001	+1.079 ± 0.043	< 0.001
SEX ➔ HorvathAgeEAA	−0.201 ± 0.040	< 0.001	−0.203 ± 0.051	< 0.001
SEX ➔ DunedinPoAm	−0.270 ± 0.039	< 0.001	−0.189 ± 0.058	0.001
SEX ➔ GrimAgeEAA	−0.500 ± 0.026	< 0.001	−0.430 ± 0.036	< 0.001
NHB ➔ DunedinPoAm	+0.192 ± 0.048	< 0.001	+0.231 ± 0.060	< 0.001
NHB ➔ HorvathAgeEAA	−0.126 ± 0.052	0.016	−0.130 ± 0.055	0.020
NHB ➔ HannumAgeEAA	−0.358 ± 0.033	< 0.001	−0.267 ± 0.038	< 0.001
HISP ➔ HorvathAgeEAA	−0.110 ± 0.045	0.014	−0.130 ± 0.070	0.063
HISP ➔ PhenoAgeEAA	+0.197 ± 0.030	< 0.001	+0.120 ± 0.047	0.011
HorvathAgeEAA ➔ DunedinPoAm	+0.136 ± 0.020	< 0.001	+0.119 ± 0.029	< 0.001
HorvathAgeEAA ➔ HannumAgeEAA	+0.433 ± 0.018	< 0.001	+0.436 ± 0.032	< 0.001
HorvathAgeEAA ➔ PhenoAgeEAA	+0.603 ± 0.015	< 0.001	+0.588 ± 0.025	< 0.001
HannumAgeEAA ➔ TELO_MEAN	−0.132 ± 0.016	< 0.001	−0.093 ± 0.023	< 0.001
PhenoAgeEAA ➔ HannumAgeEAA	+0.357 ± 0.018	< 0.001	+0.357 ± 0.028	< 0.001
PhenoAgeEAA ➔ GrimAgeEAA	+0.163 ± 0.014	< 0.001	+0.169 ± 0.020	< 0.001
GrimAgeEAA ➔ DIED	+0.421 ± 0.027	< 0.001	0.493 ± 0.037	< 0.001
DunedinPoAm ➔ GrimAgeEAA	+0.607 ± 0.014	< 0.001	+0.648 ± 0.020	< 0.001
DunedinPoAm ➔ PhenoAgeEAA	+0.269 ± 0.014	< 0.001	+0.269 ± 0.019	< 0.001
HRS 2016–2022 (*n* = 1029)				
AGE ➔ TELO_MEAN	−0.140 ± 0.031	< 0.001	−0.084 ± 0.041	0.041
GrimAgeEAA ➔ TELO_MEAN	−0.052 ± 0.031	0.089	−0.037 ± 0.037	0.32
AGE ➔ DIED	+0.944 ± 0.067	< 0.001	1.014 ± 0.078	< 0.001
SEX ➔ HorvathAgeEAA	−0.187 ± 0.063	0.003	−0.149 ± 0.074	0.046
SEX ➔ DunedinPoAm	−0.258 ± 0.062	< 0.001	−0.267 ± 0.077	0.001
SEX ➔ GrimAgeEAA	−0.563 ± 0.044	< 0.001	−0.525 ± 0.054	< 0.001
NHB ➔ DunedinPoAm	+0.347 ± 0.100	< 0.001	+0.315 ± 0.117	0.007
NHB ➔ HannumAgeEAA	−0.498 ± 0.083	< 0.001	−0.458 ± 0117	< 0.001
HISP ➔ PhenoAgeEAA	+0.138 ± 0.098	0.16	+0.110 ± 0.137	0.42
HorvathAgeEAA ➔ DunedinPoAm	+0.120 ± 0.032	< 0.001	+0.116 ± 0.037	0.002
HorvathAgeEAA ➔ HannumAgeEAA	+0.366 ± 0.026	< 0.001	+0.391 ± 0.047	< 0.001
PhenoAgeEAA ➔ HannumAgeEAA	+0.344 ± 0.027	< 0.001	+0.349 ± 0.043	< 0.001
PhenoAgeEAA ➔ GrimAgeEAA	+0.212 ± 0.023	< 0.001	+0.223 ± 0.054	< 0.001
GrimAgeEAA ➔ DIED	+0.462 ± 0.058	< 0.001	+0.442 ± 0.072	< 0.001
DunedinPoAm ➔ GrimAgeEAA	+0.541 ± 0.022	< 0.001	+0.529 ± 0.028	< 0.001
DunedinPoAm ➔ PhenoAgeEAA	+0.254 ± 0.031	< 0.001	+0.237 ± 0.040	< 0.001

Abbreviations: AGE, Baseline age; DIED, Death event (yes vs. no); DunedinPoAm, Dunedin Pace of Aging DNA methylation clock; GrimAgeEAA, Grim DNA methylation Epigenetic Age Acceleration; HannumAgeEAA, Hannum DNA methylation Age, Epigenetic Age Acceleration; HISP, Hispanic; HISP, Hispanic; HorvathAgeEAA, Horvath DNA methyalation Age, Epigenetic Age Acceleration; HRS, Health and Retirement Study; *n*, unweighted sample; NHANES, National Health and Nutrition Examination Surveys; NHB, Non‐Hispanic Black; OTHER, Other race/ethnicities; PhenoAgeEAA, Pheno DNA methylation Age Epigenetic Age Acceleration; SEX, Female (1) versus Male (0); TELO_MEAN, Mean telomere length.

^a^ Generalized structural equations models were conducted as a series of linear (most equations) and Weibull models (for the DIED outcome equation). The structure of each model was determined based on the three‐parent limit solution from ABNs for NHANES and HRS cohorts. Continuous variables are entered as standardized z‐scores, while binary variables are entered as 1 versus 0.

^b^ Model 1 was conducted without adjustment for sampling design complexity and thus assuming a simple random sample.

^c^ Model 2 adjusted for sampling design complexity by including sampling weights, PSU and strata that were most appropriate for each cohort.

^d^ *p*‐value for null hypothesis that path coefficient *β* = 0.

## 4 Discussion

### 4.1 Summary of Findings

The present study uses ABNs to examine the link between DNAm clocks, telomere length, and mortality risk in three US populations (NHANES, HRS and HANDLS). Validation was accomplished by additional analyses with HANDLS data. Among key findings, epigenetic clocks, particularly GrimAgeEAA, HannumAgeEAA, and DunedinPoAM (or DunedinPACE in HANDLS), demonstrated stronger and consistent associations with mortality risk compared to telomere length. ABNs revealed nuanced relationships, with age and GrimAgeEAA consistently predicting mortality risk across NHANES and HRS. Based on GSEM models selected from ABNs, sex had a significant inverse association with GrimAgeEAA in both NHANES and HRS samples, suggesting that females generally exhibit lower biological aging as measured by GrimAgeEAA compared to males. GrimAgeEAA, in turn, strongly predicted mortality (Ln(Hazard Ratio) or LnHR, *β* ± SE of +0.476 ± 0.0393 in NHANES and +0.511 ± 0.0775 in HRS). A similar but weaker pattern was observed for DunedinPoAm in both cohorts. NHB participants showed faster biological aging as measured by DunedinPoAm compared to NHW participants, with less consistencies across cohorts with respect to other racial/ethnic contrasts.

### 4.2 Previous Studies

#### 4.2.1 Telomere Length, Morbidity, and Mortality

Multiple studies, with varying findings, have investigated the association between TL and mortality outcomes, including all‐cause and disease‐specific mortality (Adegunsoye et al. 2023; Arbeev et al. 2020; Chen et al. 2023; Cheng et al. 2021; Gao et al. 2023; Gao et al. 2020; Herrmann and Herrmann 2020; Huang et al. 2019; Jian et al. 2024; Jiang et al. 2023; Lan et al. 2022; Mons et al. 2017; Premuzic et al. 2024; Schneider et al. 2022; Shen et al. 2020; Wang et al. 2024; Wang et al. 2018; Xiong et al. 2023; Yeap et al. 2021; Zhan et al. 2018). Various cohorts such as the UK Biobank, NHANES, and other multi‐cohort studies with sample sizes ranging from a few hundred to hundreds of thousands of participants have been used for these studies. These studies have adjusted models for the effects of different covariates, including age, sex, ethnicity, socioeconomic status, and health‐related factors. Overall, shorter TL was generally associated with increased mortality risk. For example, in a study involving data from three cohorts of European ancestry, a 1‐kilobase decrease in LTL was associated with a HR of 1.34 (95% CI, 1.21–1.47) for all‐cause mortality and 1.53 (95% CI, 1.32–1.77) for cancer‐specific mortality. However, some studies also reported non‐significant findings or unexpected associations between TL and mortality (Chen et al. 2023; Gao et al. 2023). Furthermore, other studies indicated that frailty mediated part of the TL‐mortality relationship (Jian et al. 2024), and TL had varying impacts on mortality risk depending on comorbidities like type 2 diabetes and cardiovascular disease (Cheng et al. 2021; Xiong et al. 2023). A systematic review and meta‐analysis further confirmed the link between TL and mortality, demonstrating a higher hazard ratio for individuals with shorter telomeres, with sex‐ and ethnicity‐based variations (Wang et al. 2018). Our findings suggest that telomere length may have cohort‐specific associations with mortality, showing expected inverse associations in NHANES but inconsistent or unexpected patterns in HRS and HANDLS. In contrast, epigenetic clocks—especially GrimAgeEAA and PhenoAgeEAA—were more robust and consistent predictors of mortality across cohorts. Sex and race/ethnicity influenced aging trajectories: Women had slower biological aging and lower mortality risk, while racial patterns varied by metric. These results support the superiority of epigenetic clocks over telomere length in predicting mortality and highlight the importance of considering demographic and cohort context in aging research.

#### 4.2.2 Epigenetic Clocks, Morbidity, and Mortality

Recently, molecular targets as clinical biomarkers and as ways to predict age‐related diseases and mortality have garnered interest. The use of epigenetic biomarkers of aging known as epigenetic clocks using DNAm metrics has historically provided accurate estimations of aging at various life stages (Fransquet et al. 2019; Horvath and Raj 2018). A meta‐analysis of 23 articles reported a 5‐year increase in DNAm age‐related to an 8%–15% increased mortality risk (Fransquet et al. 2019). In another study, intrinsic EAA Hannum and age acceleration Grim predicted oropharyngeal cancer mortality (Beynon et al. 2022). Additionally, Horvath, Hannum, or Grim EAA predicted cancer mortality and Grim EAA also predicted cardiovascular mortality (Beynon et al. 2022; Mendy and Mersha 2024; Perna et al. 2016). These results reveal the significance of epigenetic markers' relationships with morbidity and mortality outcomes. Despite lacking an evaluation of cause‐specific mortality, our study generally replicated those prior findings from various distinctive cohorts and varied types of analytic approaches. Specifically, our findings support prior research showing that EAA measures, particularly GrimAge and Hannum, are associated with increased risk of death from cancer and cardiovascular disease. The robust performance of these clocks across diverse cohorts reinforces their potential as clinical biomarkers of aging and mortality.

#### 4.2.3 Association of Telomere Length With Epigenetic Clocks

Research on the links among epigenetic clocks, telomere length, and other facets of aging has been conducted. According to Vetter et al. (2022), although their association with telomere length and functional capability is complicated and varies depending on the particular clock utilized, epigenetic clocks are linked with chronological age (Vetter et al. 2022). Limited cross‐sectional correlations between telomere length and epigenetic clocks were reported by Pearce et al. (2022), implying that these indicators could represent several facets of biological aging (Pearce et al. 2022). Suggesting that these two biomarkers may contribute separately to the knowledge of biological aging, Banszerus et al. (2019) did not uncover a clear correlation between relative telomere length and epigenetic age acceleration (Banszerus et al. 2019). While both metrics are independently linked with chronological age, Marioni et al. (2018) found that only the epigenetic clock significantly predicted death (Marioni et al. 2018). Chen et al. (2017) investigated the relationship between leukocyte telomere length (LTL) and extrinsic epigenetic age acceleration (EEAA), a DNA methylation‐based biomarker predictive of mortality (Chen et al. 2017). Analyzing data from over 2500 participants across three cohorts, they found that shorter LTL correlated with higher EEAA (Chen et al. 2017). This association was linked to immune cell composition: Individuals with more memory CD8+ T cells and fewer naive CD8+ T cells exhibited both shorter telomeres and older epigenetic age (B. H. Chen et al. 2017). The findings suggest that LTL reflects immune system aging and contributes to EEAA's predictive power for mortality (Chen et al. 2017).

These findings highlight the complexity of biological aging and suggest that no single biomarker can fully capture its multifaceted nature. Both telomere length and epigenetic clocks provide valuable but distinct information about aging, with limited overlap between them. In our study, only HannumAgeEAA showed a weak inverse correlation with telomere length in NHANES, reinforcing evidence from previous research that these measures reflect different biological processes. Therefore, a comprehensive understanding of aging and its links to mortality may require a combination—or battery—of biomarkers. Continued research is essential to clarify how these markers can best be used to predict health outcomes.

#### 4.2.4 Role of Biological Aging in Explaining Differences in Mortality

Studies have shown that the epigenetic clock and telomere length are correlated with chronological age and mortality, but the epigenetic clock is a more robust predictor of mortality (Hillary et al. 2020; Horvath et al. 2016; Marioni et al. 2018). Epigenetic aging rates are significantly associated with sex and race/ethnicity. In general, men have shown higher aging rates than women across various tissues, with varying differences shown across racial and ethnic groups depending on the clock in question (Horvath et al. 2016). Epigenetic measures of aging, such as DNAm GrimAge, are associated with the incidence of diseases like COPD, type 2 diabetes, and ischemic heart disease (Hillary et al. 2020). These studies highlight the importance of epigenetic clocks as biomarkers for aging and health outcomes.

#### 4.2.5 Biological Mechanisms Behind Relationships Among Telomere Length, Epigenetic Clocks, and Mortality

Many mechanisms may affect telomere length, epigenetic clocks, and mortality. They can be influenced by genetic factors but also by lifestyle and environmental factors. For example, telomere length and epigenetic age acceleration are both influenced by lifestyle factors such as physical activity, diet, smoking, and other environmental exposures (Oblak et al. 2021; Vaiserman and Krasnienkov 2020). Yet, it appears that some biological mechanisms that affect telomere length and epigenetic aging do so differently (Oblak et al. 2021; Vaiserman and Krasnienkov 2020). These differences may lie in that telomere length is affected by cell division and may reflect more of a “mitotic clock” (Oblak et al. 2021; Vaiserman and Krasnienkov 2020). Epigenetic clock predictability relies heavily on the measures that they were trained upon (Oblak et al. 2021; Vaiserman and Krasnienkov 2020). These vary from chronological age (Hannum, Horvath) to mortality (GrimAge) to phenotypic age (PhenoAge, DunedinPoAm, DunedinPACE) (Belsky et al. 2020; Belsky et al. 2022; Oblak et al. 2021; Vaiserman and Krasnienkov 2020). Therefore, different aspects of biological aging may be indicative of these different measures (Oblak et al. 2021; Vaiserman and Krasnienkov 2020). However, GWAS studies have indicated that variants associated with telomerase reverse transcriptase (TERT), the enzyme that elongates telomeres, and longer TL are associated with higher intrinsic age acceleration (Lu et al. 2018). This is unexpected but experiments in cells grown in vitro suggested that cell proliferation over time is associated with an increase in DNAm age (Lu et al. 2018). Therefore, these data indicate a linear relationship between telomere length and DNAm age in cells grown in vitro, but this may not be reflective of biological aging in humans, which is more complex and dynamic.

Consequently, our findings echo sex and racial/ethnic patterns reported in previous research where women exhibited slower biological aging, and non‐Hispanic Black adults had mixed aging profiles depending on the metric used. More specifically, non‐Hispanic Black (NHB) participants showed distinct patterns across biological aging measures. In both NHANES and HRS, NHB individuals had significantly higher DunedinPoAm scores (β = +0.192 in NHANES; β = +0.347 in HRS) and significantly lower HannumAgeEAA values (β = −0.358 in NHANES; β = −0.498 in HRS) compared to non‐Hispanic Whites. In the HRS cohort, NHB participants also had shorter telomere length, a pattern not observed in NHANES or HANDLS. No significant associations between race and HorvathAgeEAA or GrimAgeEAA were observed in either cohort. These findings indicate that NHB status was associated with faster aging by DunedinPoAm and slower aging by HannumAgeEAA across cohorts, with telomere length differences evident only in HRS.

#### 4.2.6 Comparisons of Various Epigenetic Clocks

As the various epigenetic clocks were trained on different measures, they capture different aspects of biological epigenetic aging. In our data, we find that, in NHANES and HRS cohorts, there was a correlation between Horvath and Hannum EAA and between GrimAge EAA and DunedinPoAM. In the HANDLS cohort, HorvathAgeEAA and HannumAgeEAA were correlated and strongly only weakly correlated with the DunedinPACE. These data are consistent with the fact that the Horvath and Hannum clocks are considered “first generation” and trained on chronological age, whereas the second‐ and third‐generation clocks, PhenoAge and GrimAge, DunedinPoAM, and DunedinPACE were trained on phenotypes or mortality (Belsky et al. 2020; Belsky et al. 2022; Oblak et al. 2021; Vaiserman and Krasnienkov 2020). Therefore, calculating all four epigenetic clocks in three different cohorts as we have done here yields important and novel information related to these measures and their relationships to mortality and telomere length in different cohorts (Belsky et al. 2020; Belsky et al. 2022; Oblak et al. 2021; Vaiserman and Krasnienkov 2020).

#### 4.2.7 GWAS and MR of Epigenetic Clocks and Telomere Length

Recent advances in Genome‐Wide Association Studies (GWAS) and Mendelian Randomization (MR) have illuminated genetic and epigenetic mechanisms underlying aging. A GWAS of over 40,000 individuals identified 137 loci associated with DNA methylation‐based aging biomarkers, implicating genes tied to lipid metabolism, immune function, and longevity (McCartney et al. 2021). MR analyses suggest causal links between smoking and insomnia with telomere shortening, while physical activity may preserve telomere length (Chen et al. 2024). MR studies have also identified a potential causal role of GrimAge acceleration in colorectal cancer risk (Morales Berstein et al. 2022) and showed that variations in white blood cell counts significantly affect age acceleration metrics like PhenoAge and GrimAge (Sun et al. 2024). However, other studies found no causal relationship between epigenetic age acceleration and pulmonary vascular diseases (Tong et al. 2024). These findings underscore both the promise and limitations of genetic and epigenetic research in aging, highlighting the need for cautious interpretation across diverse health outcomes.

### 4.3 Strengths and Limitations

Several strengths can be noted for this study. First, it focuses on the relationship between biological aging and mortality risk in US adults, using three distinct datasets: NHANES, HRS, and HANDLS. It incorporates telomere length and multiple measures of EAA for a comprehensive evaluation of biological aging and its association with mortality risk. The use of ABNs offers a robust methodological framework for uncovering complex, probabilistic relationships among biological aging markers, social determinants, and mortality while accommodating confounding and mediation effects. The study also uses longitudinal mortality data linked to biomarkers to investigate long‐term health outcomes. Advanced statistical methods, such as LASSO regression for variable selection, Cox models for mortality risk, and GSEM for pathway validation, enhance the rigor and depth of the analyses.

However, the study has limitations such as measurement variability across datasets, residual confounding, cross‐sectional biomarker data, potential selection bias, complexity of Bayesian networks, limited statistical power to study cause‐specific mortality risk in relation to biological aging metrics or to stratify results by sex and race, various sample‐specific biases, and computational demands of ABN. These limitations may limit the generalizability of findings. In fact, a key limitation of our study is that widely used epigenetic clocks such as Horvath and DunedinPoAm (or DunedinPACE) were primarily developed using data from individuals of European ancestry. As a result, their accuracy and validity in estimating epigenetic age acceleration (EAA) among individuals of non‐European ancestry, including Black and Hispanic populations, may be reduced. This raises concerns about potential biases in age‐related biomarker estimates and the generalizability of findings across diverse populations. Several studies have highlighted ancestry‐related differences in DNA methylation patterns that may influence clock performance (Horvath et al. 2016). In a recent study by Shen et al. that uses HANDLS data, DunedinPACE was associated with accelerated aging in below poverty White participants but scores were similar with above and below poverty African Americans (B. Shen et al. 2023). Efforts to develop and validate ancestry‐inclusive or ancestry‐specific clocks are ongoing, and future studies should prioritize diverse cohorts to improve the equity and utility of these biomarkers. Despite these limitations, the use of multiple cohorts and sophisticated analysis methods for this study offers unique insights into aging biology and could trigger additional research in this area.

## 5 Conclusions

In summary, epigenetic clocks, particularly GrimAgeEAA, are stronger and consistent predictors of mortality risk compared to telomere length across different US cohorts. These findings highlight the potential of advanced biomarkers of biological aging to enhance our understanding of differences in mortality risk across populations. Additive Bayesian networks further revealed complex relationships between biological aging markers, demographics, and mortality risk, underscoring the role of these biomarkers in capturing nuanced pathways underlying disparities in aging and survival.

## Author Contributions

May A. Beydoun: Conceptualization, data curation, statistical analysis, supervision, data acquisition, methodology, validation, write‐up of manuscript, revision of the manuscript. Nicole Noren Hooten: Conceptualization, data acquisition, methodology, resources, validation, write‐up of manuscript, revision of the manuscript. Nigus G. Asefa: Conceptualization, validation, methodology, write‐up of manuscript, revision of the manuscript. Michael F. Georgescu: Conceptualization, validation, write‐up of manuscript, revision of the manuscript. Minkyo Song, Sri Banerjee, Jagdish Khubchandani: Conceptualization, write‐up of manuscript, revision of the manuscript. Hind A. Beydoun: Conceptualization, data curation, write‐up of manuscript, revision of the manuscript. Osorio Meirelles: Conceptualization, methodology, write‐up of manuscript, revision of the manuscript. Lenore J. Launer: Conceptualization, supervision, methodology, resources, write‐up of manuscript, revision of the manuscript. Michele K. Evans: Conceptualization, supervision, data acquisition, resources, write‐up of manuscript, revision of the manuscript. Alan B. Zonderman: Conceptualization, data curation, supervision, data acquisition, resources, write‐up of manuscript, revision of the manuscript.

## Conflicts of Interest

The authors declare no conflicts of interest.

## Supporting information


**Figure S1.** Participant flowcharts for NHANES, HRS, and HANDLS samples.

**Figure S2.** LASSO findings for NHANES, HRS, and HANDLS samples: TELO_MEAN versus epigenetic clock metrics.

**Figure S3.** ABN findings using discrete time hazards models, for 1 and 2 parents/child limits (A) NHANES 1999–2002, follow‐up till 2019.

**Figure S4.** Additive Bayesian network (ABN) model fit across number of parents per child in two US cohorts.

Appendix S1.

## Data Availability

The study protocol (09‐AG‐N248) of HANDLS received approval from the National Institute on Environmental Health Sciences' Institutional Review Board (IRB) of the National Institutes of Health (NIH). Upon request, data can be made available to researchers with approved proposals, after they have agreed to confidentiality as required by our IRB. Policies are publicized on: https://handls.nih.gov. Data access request can be sent to principal investigators (PI) or the study manager, Jennifer Norbeck at norbeckje@mail.nih.gov. These data are owned by the National Institute on Aging at the NIH. The PIs have made those data restricted to the public for two main reasons: “(1) The study collects medical, psychological, cognitive, and psychosocial information on racial and poverty differences that could be misconstrued or willfully manipulated to promote racial discrimination; and (2) Although the sample is fairly large, there are sufficient identifiers that the PIs cannot guarantee absolute confidentiality for every participant as we have stated in acquiring our confidentiality certificate.” (Beydoun, Weiss, et al. 2019) NHANES and HRS data are publicly available at: https://www.cdc.gov/nchs/nhanes/index.htm and https://hrs.isr.umich.edu/about, respectively. Code and Output can be obtained from the corresponding author at baydounm@mail.nih.gov and will be made available on github at: https://github.com/baydounm/HRS_NHANES_HANDLS_TLEPIGENMORT/tree/main.
