# Supplementary material for: Telomere Length, Epigenetic Age Acceleration, and Mortality Risk in US Adult Populations: An Additive Bayesian Network Analysis
Source: Aging Cell. 2025 Jul 6;24(9):e70159. doi: 10.1111/acel.70159 (PMC12419851; doi:10.1111/acel.70159)
Supplement: Supplementary file 1 — Figure S1. Participant flowcharts for NHANES, HRS, and HANDLS samples. [file ACEL-24-e70159-s005.pdf]

FIGURE S1. Participant flowcharts for NHANES, HRS and HANDLS samples

(A) NHANES 1999-2002

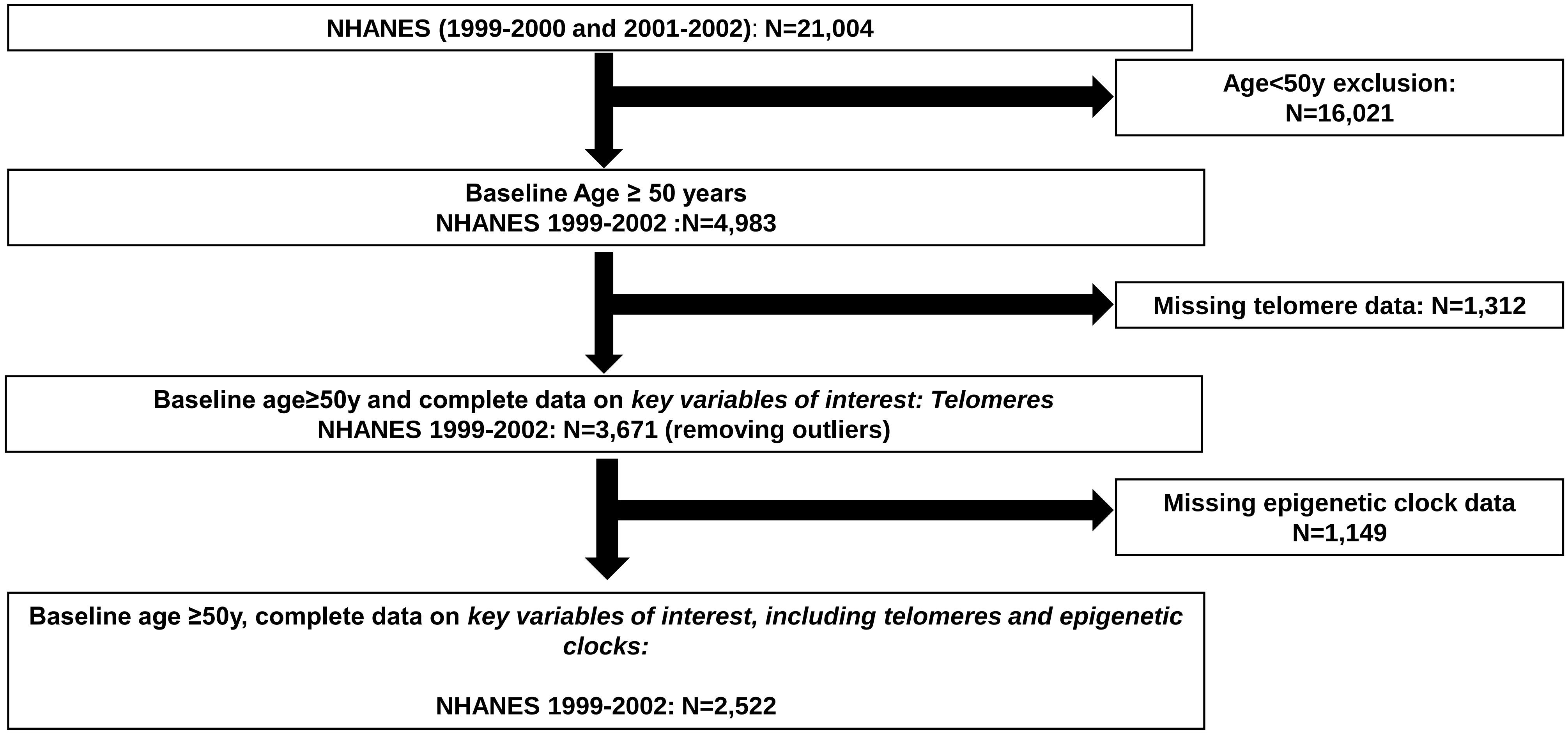

(B) HRS 2008 (telomeres) and 2016 (epigenetic clocks)

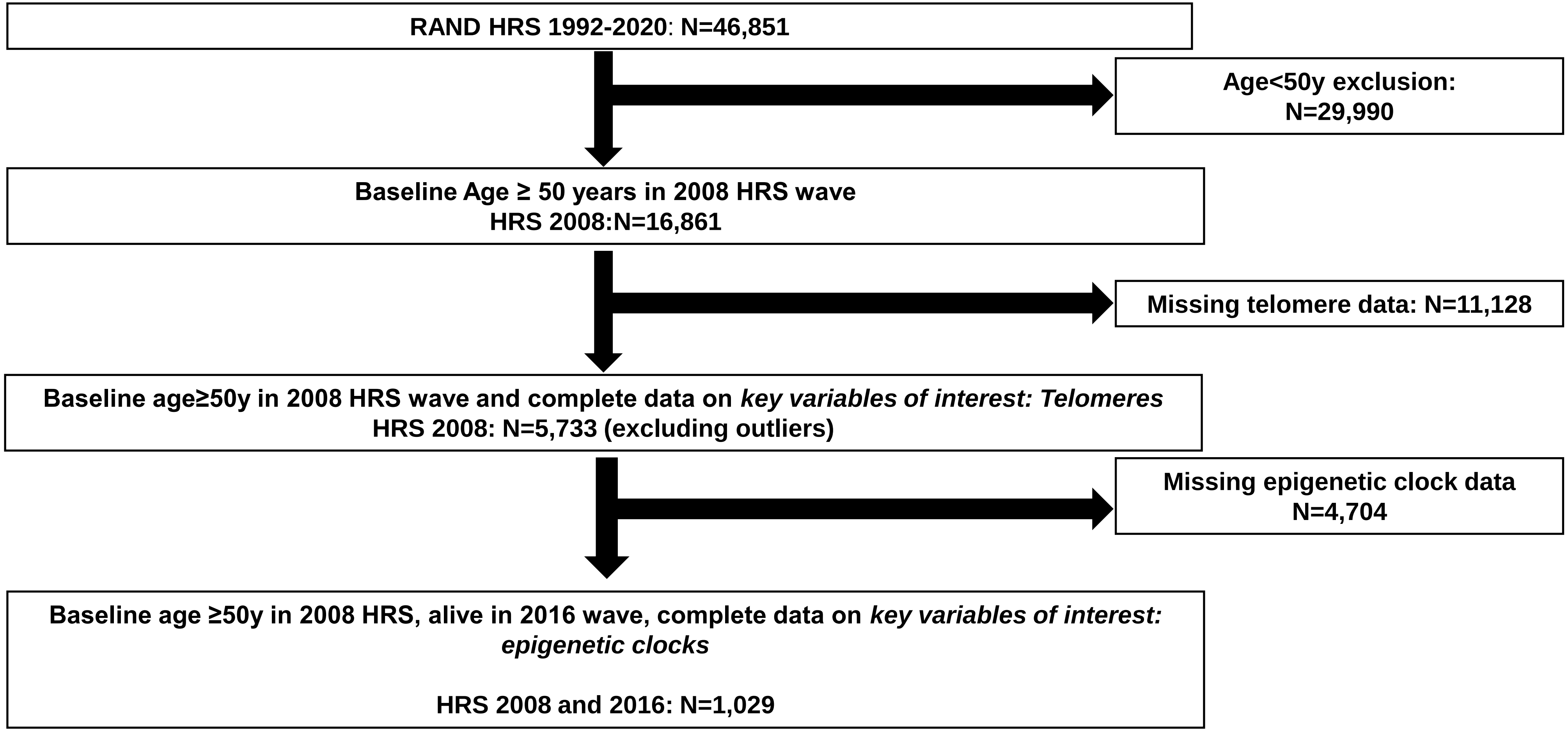

(C) HANDLS 2004-2009

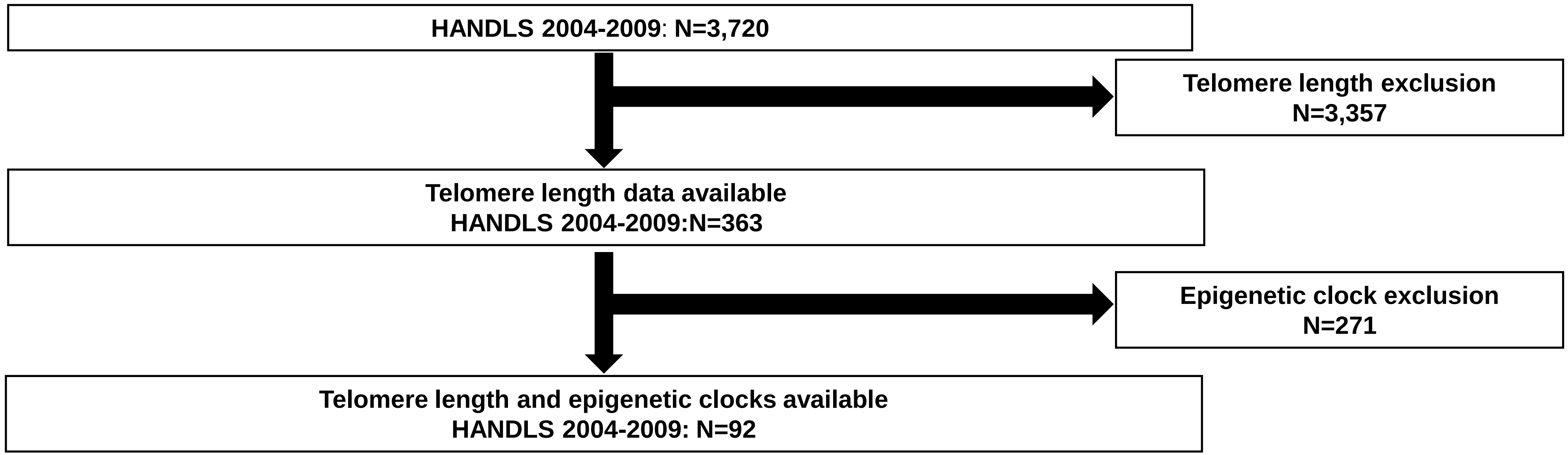

Notes: For all 3 cohorts, the largest available sample with all key variables of interest was selected, without any further exclusions. In the HANDLS study, part of the analysis was carried out on the largest sample with epigenetic clocks (n=470) and telomere length(n=363), while other parts of the analysis were completed on the final sample with both data available (n=92).

Abbreviations: HANDLS=Healthy Aging in Neighborhoods of Diversity across the Life Span; HRS=Health and Retirement Study; NHANES=National Health and Nutrition Examination Surveys.
