# Supplementary material for: Telomere Length, Epigenetic Age Acceleration, and Mortality Risk in US Adult Populations: An Additive Bayesian Network Analysis
Source: Aging Cell. 2025 Jul 6;24(9):e70159. doi: 10.1111/acel.70159 (PMC12419851; doi:10.1111/acel.70159)
Supplement: Supplementary file 2 — Figure S2. LASSO findings for NHANES, HRS, and HANDLS samples: TELO_MEAN versus epigenetic clock metrics. [file ACEL-24-e70159-s002.pdf]

FIGURE S2. LASSO findings for NHANES, HRS and HANDLS samples: TELO_MEAN vs. epigenetic clock metrics

(A) NHANES 1999-2002

	cvTELOMEAN	minBICTELOMEAN	adaptiveTELOMEAN
AGE	x	x	x
zHannumAgeEAA	x	x	x
zHorvathAgeEAA	x	x	x
SEX	x	x	x
zPhenoAgeEAA	x	x	x
RACE			
3	x	x	x
2	x	x	x
zGrimAgeMortEAA	x	x	x
RACE			
1	x	x	x
_cons	x	x	x
RACE			
0	e	e	e

Legend:
b - base level
e - empty cell
o - omitted
x - estimated

Postselection coefficients

Name	sample_L~O	MSE	R-squared	Obs
cvTELOMEAN				
	1	.6975822	0.0951	1,272
	2	.6294661	0.1252	1,258
minBICTELOMEAN				
	1	.6975822	0.0951	1,272
	2	.6294661	0.1252	1,258
adaptiveTELOMEAN				
	1	.6975822	0.0951	1,272
	2	.6294661	0.1252	1,258

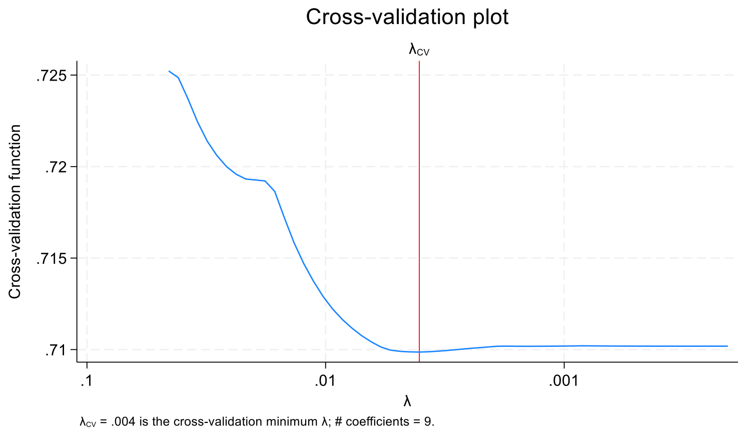

Source	SS	df	MS
Model	217.783806	9	24.1982006
Residual	1651.28175	2,512	.657357385
Total	1869.06556	2,521	.741398476

Number of obs	=	2,522
F(9, 2512)	=	36.81
Prob > F	=	0.0000
R-squared	=	0.1165
Adj R-squared	=	0.1134
Root MSE	=	.81078

ztelomean_no_~s	Coefficient	Std. err.	t	P>|t|	[95% conf.	interval]
AGE	-.0220915	.0016263	-13.58	0.000	-.0252806	-.0189023
SEX	.1242713	.0350653	3.54	0.000	.0555114	.1930312
RACE						
1	.1094472	.0444606	2.46	0.014	.0222641	.1966303
2	-.0641211	.0382083	-1.68	0.093	-.1390441	.0108018
3	-.0557036	.093624	-0.59	0.552	-.2392917	.1278845
zHorvathAgeEAA	.1377052	.0237484	5.80	0.000	.0911367	.1842736
zHannumAgeEAA	-.1566227	.0242271	-6.46	0.000	-.2041298	-.1091155
zPhenoAgeEAA	-.0557877	.0240391	-2.32	0.020	-.1029261	-.0086493
zGrimAgeMortEAA	-.0377851	.019085	-1.98	0.048	-.0752092	-.0003611
_cons	.8873628	.1235566	7.18	0.000	.6450796	1.129646

(B) HRS 2008 (telomeres) and 2016 (epigenetic clocks)

	cvTELOMEAN	minBICTELOMEAN	adaptiveTELOMEAN
AGE	x	x	x
RACE_ETHN			
3	x	x	x
4	x	x	x
2	x	x	x
SEX	x	x	x
RACE_ETHN			
1	e	e	e
_cons	x	x	x

Legend:
b - base level
e - empty cell
o - omitted
x - estimated

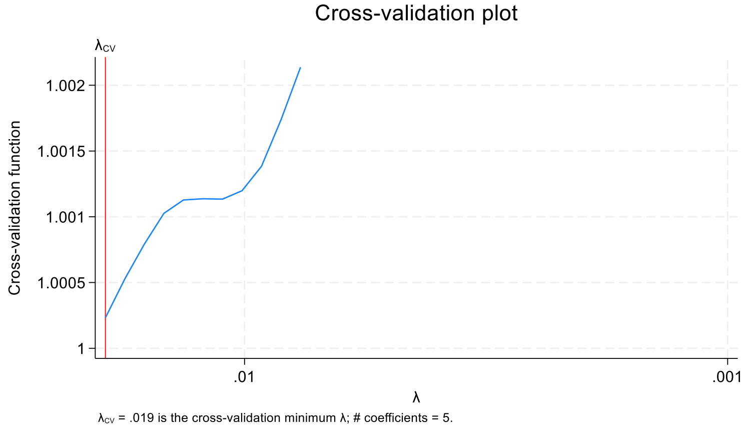

Postselection coefficients

Name	sample_L~O	MSE	R-squared	Obs
cvTELOMEAN				
	1	.9663366	0.0365	515
	2	.9872245	0.0075	514
minBICTELOMEAN				
	1	.9663366	0.0365	515
	2	.9872245	0.0075	514
adaptiveTELOMEAN				
	1	.9663366	0.0365	515
	2	.9872245	0.0075	514

Source	SS	df	MS
Model	27.9090007	5	5.58180014
Residual	1000.091	1,023	.977606058
Total	1028	1,028	.999999999

Number of obs	=	1,029
F(5, 1023)	=	5.71
Prob > F	=	0.0000
R-squared	=	0.0271
Adj R-squared	=	0.0224
Root MSE	=	.98874

ztelomean_~s	Coefficient	Std. err.	t	P>|t|	[95% conf.	interval]
AGE	-.0157022	.0037567	-4.18	0.000	-.0230739	-.0083305
SEX	.0639351	.0631088	1.01	0.311	-.0599025	.1877727
RACE_ETHN						
2	.171723	.1025549	1.67	0.094	-.0295191	.372965
3	.1907079	.1018118	1.87	0.061	-.0090758	.3904917
4	.2065721	.2250466	0.92	0.359	-.2350336	.6481779
_cons	1.090023	.2884124	3.78	0.000	.5240752	1.65597

(C) HANDLS 2004-2009

	cvTELOMEAN	minBICTELOMEAN	adaptiveTELOMEAN
AGE	x	x	x
RACE			
1	x	x	x
SEX	x	x	x
RACE			
0	e	e	e
_cons	x	x	x

Legend:
- b - base level
- e - empty cell
- o - omitted
- x - estimated

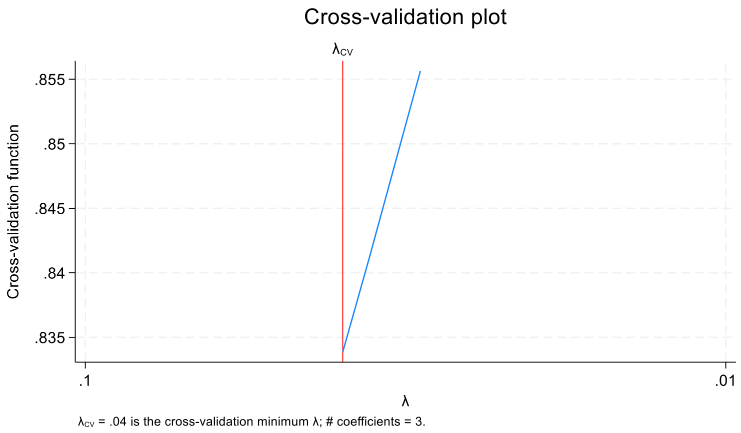

Postselection coefficients

Name	sample_L~O	MSE	R-squared	Obs
cvTELOMEAN				
	1	.6889135	0.0814	50
	2	1.269283	-0.0560	42
minBICTELOMEAN				
	1	.6889135	0.0814	50
	2	1.269283	-0.0560	42
adaptiveTELOMEAN				
	1	.6889135	0.0814	50
	2	1.269283	-0.0560	42

Source	SS	df	MS
Model	2.29164844	3	.763882814
Residual	86.1910677	88	.979443951
Total	88.4827161	91	.97233754

Number of obs	=	92
F(3, 88)	=	0.78
Prob > F	=	0.5083
R-squared	=	0.0259
Adj R-squared	=	-0.0073
Root MSE	=	.98967

ztelomean_~s	Coefficient	Std. err.	t	P>|t|	[95% conf.	interval]
AGE	-.0096979	.0131873	-0.74	0.464	-.0359049	.016509
SEX	-.2153838	.2118528	-1.02	0.312	-.6363966	.2056291
1.RACE	.2086122	.2297522	0.91	0.366	-.2479721	.6651964
_cons	.3771274	.6715238	0.56	0.576	-.957385	1.71164

Notes: LASSO is a regression regularization method that improves prediction accuracy and interpretability by shrinking coefficients to zero. It comes in three variants: cross-validated LASSO, adaptive LASSO, and minimum Bayesian Information Criterion LASSO. In this study, it is implemented using Stata. Details are provided in **Appendix IV**. Unweighted sample sizes were n=2,522 for NHANES, n=1,029 for HRS and n=92 for HANDLS.

Abbreviations: DunedinPoAm=Dunedin Pace of Aging DNA methylation clock; GrimAgeEAA=Grim DNA methylation Epigenetic Age Acceleration; HANDLS=Healthy Aging in Neighborhoods of Diversity across the Life Span; HannumAgeEAA=Hannum DNA methylation Age, Epigenetic Age Acceleration; HorvathAgeEAA=Horvath DNA methyalation Age, Epigenetic Age Acceleration; HRS=Health and Retirement Study; LASSO=Least Absolute Shrinkage Selection Operator; NHANES=National Health and Nutrition Examination Surveys; PhenoAgeEAA=Pheno DNA methylation Age Epigenetic Age Acceleration; TELO_MEAN=Mean telomere length; z=standardized z-score.
