# Supplementary material for: Telomere Length, Epigenetic Age Acceleration, and Mortality Risk in US Adult Populations: An Additive Bayesian Network Analysis
Source: Aging Cell. 2025 Jul 6;24(9):e70159. doi: 10.1111/acel.70159 (PMC12419851; doi:10.1111/acel.70159)
Supplement: Supplementary file 3 — Figure S3. ABN findings using discrete time hazards models, for 1 and 2 parents/child limits (A) NHANES 1999–2002, follow‐up till 2019. [file ACEL-24-e70159-s001.pdf]

FIGURE S3. ABN findings using discrete time hazards models, for 1 and 2 parents/child limits

(A) NHANES 1999-2002, follow-up till 2019

1 parent/child

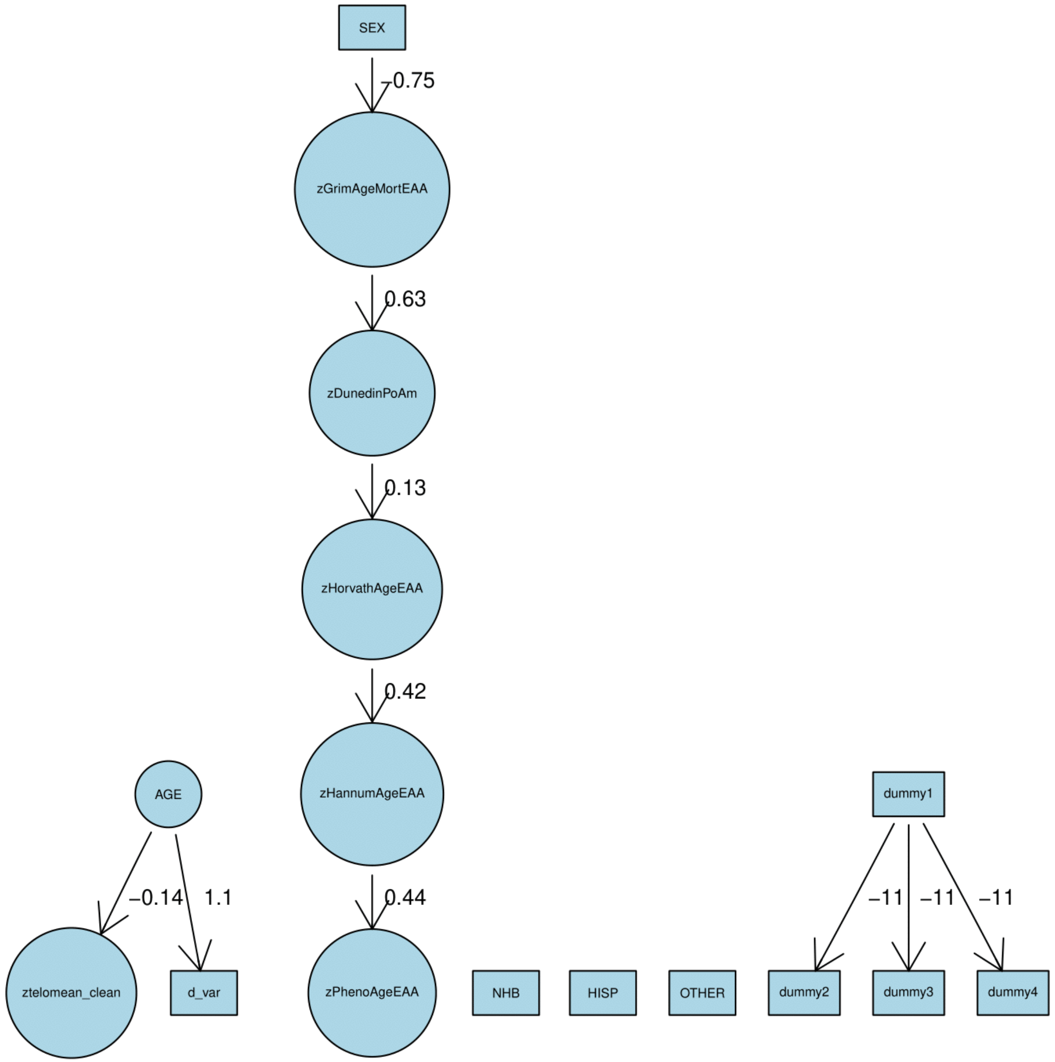

2 parents/child

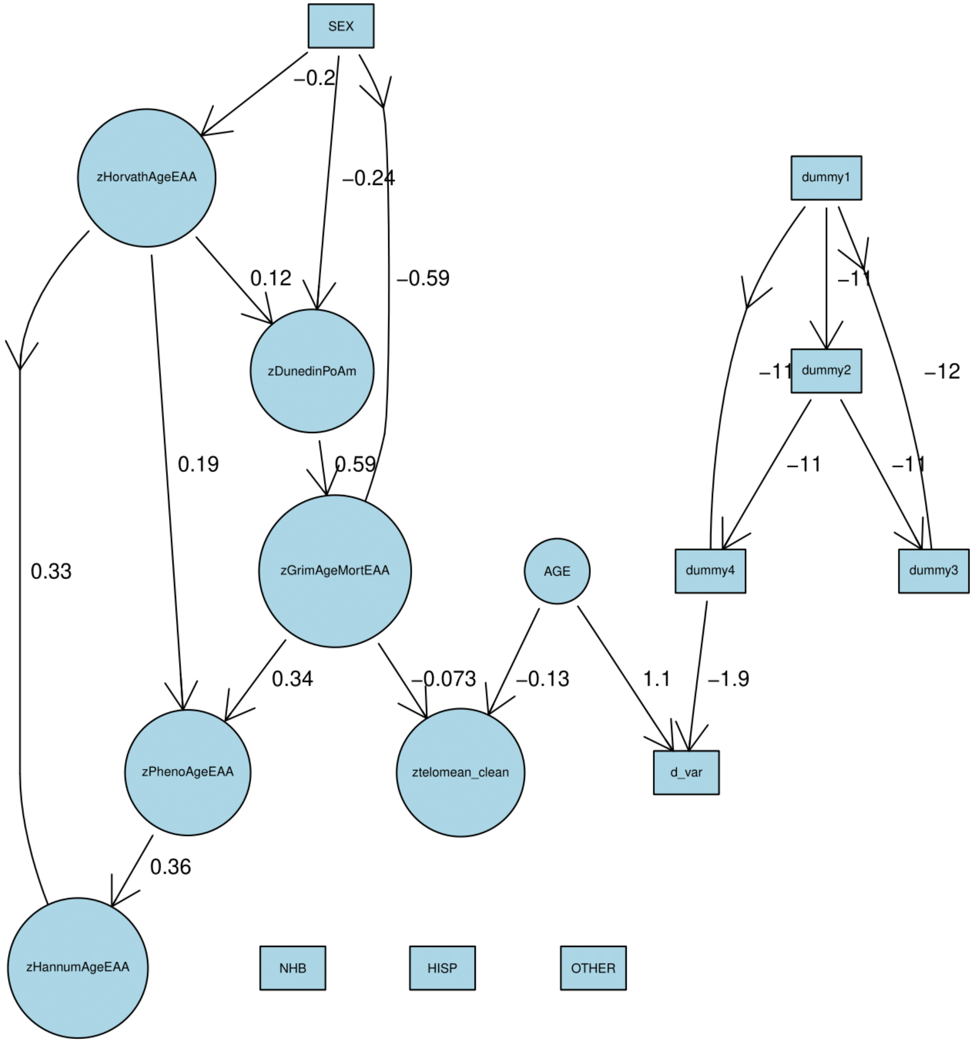

(B) HRS 2008 (telomeres) and 2016 (epigenetic clocks) follow-up till 2022

1 parent/child

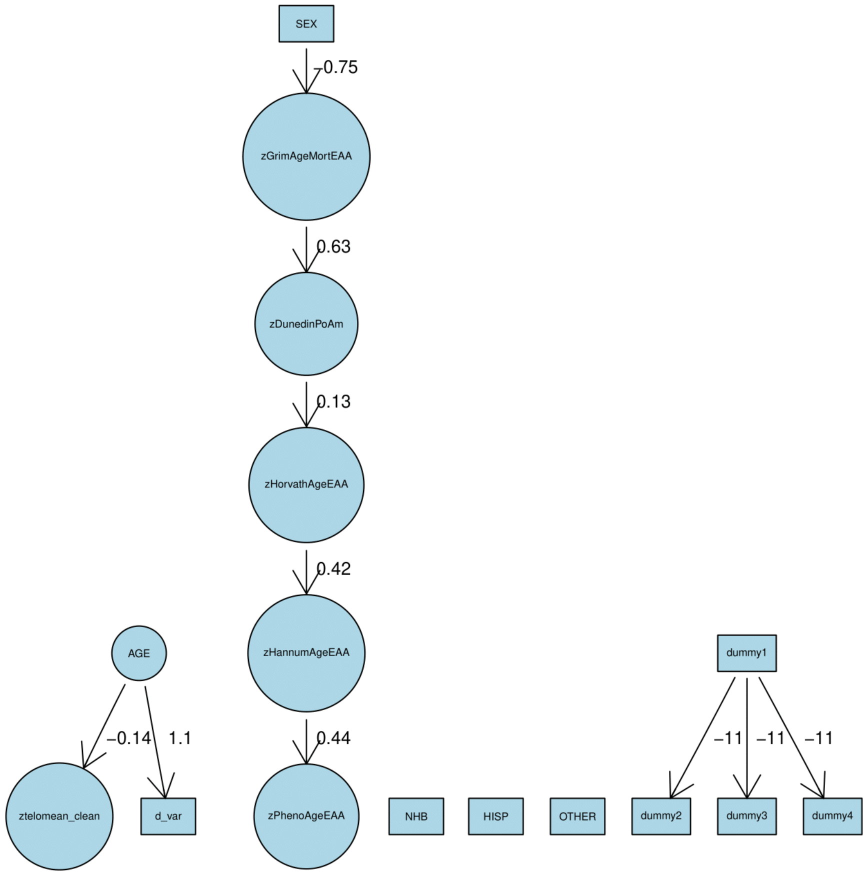

2 parents/child

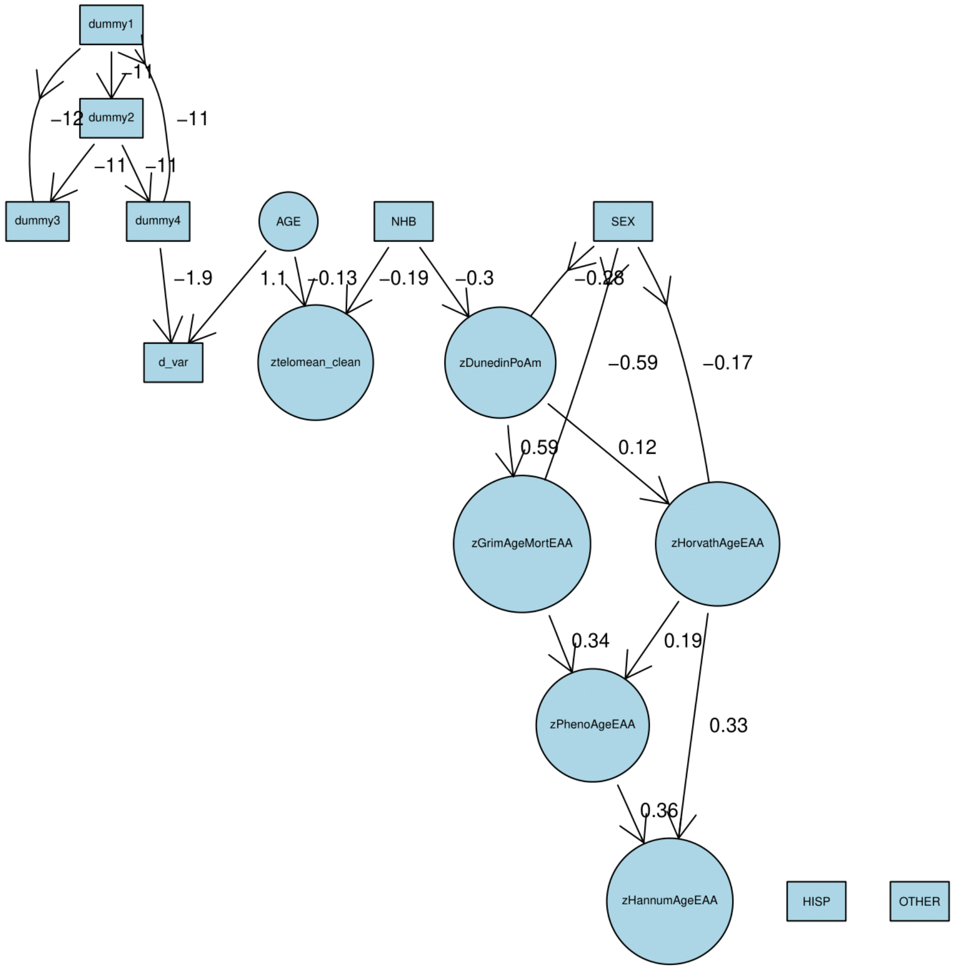

Notes: Details for R code used for this analysis described in **Appendix VI** and provided on github. This code provides a comprehensive pipeline for conducting ABN analysis, including installation, data preprocessing, constraint specification, model fitting, and iterative optimization. It involves installing R versions 4.4 or higher, data preparation, data wrangling, defining variable groups, setting constraints, optimizing across parent limits, building the additive Bayesian network, and generating visual representations. The optimal number of parents of a child is determined based on levelling off the log marginal likelihood and desired complexity between key variables. Unweighted sample sizes were n=2,522 for NHANES and n=1,029 for HRS.

Abbreviations: DunedinPoAm=Dunedin Pace of Aging DNA methylation clock; GrimAgeEAA=Grim DNA methylation Epigenetic Age Acceleration; HannumAgeEAA=Hannum DNA methylation Age, Epigenetic Age Acceleration; HorvathAgeEAA=Horvath DNA methyalation Age, Epigenetic Age Acceleration; HRS=Health and Retirement Study; NHANES=National Health and Nutrition Examination Surveys; PhenoAgeEAA=Pheno DNA methylation Age Epigenetic Age Acceleration; TELO_MEAN=Mean telomere length; z=standardized z-score.
