# Supplementary material for: Telomere Length, Epigenetic Age Acceleration, and Mortality Risk in US Adult Populations: An Additive Bayesian Network Analysis
Source: Aging Cell. 2025 Jul 6;24(9):e70159. doi: 10.1111/acel.70159 (PMC12419851; doi:10.1111/acel.70159)
Supplement: Supplementary file 4 — Figure S4. Additive Bayesian network (ABN) model fit across number of parents per child in two US cohorts. [file ACEL-24-e70159-s003.pdf]

FIGURE S4. Additive Bayesian Network (ABN) Model Fit Across Number of Parents per Child in Two U.S. Cohorts

(A) NHANES 1999-2019

Model fit for 1-3 parents/child

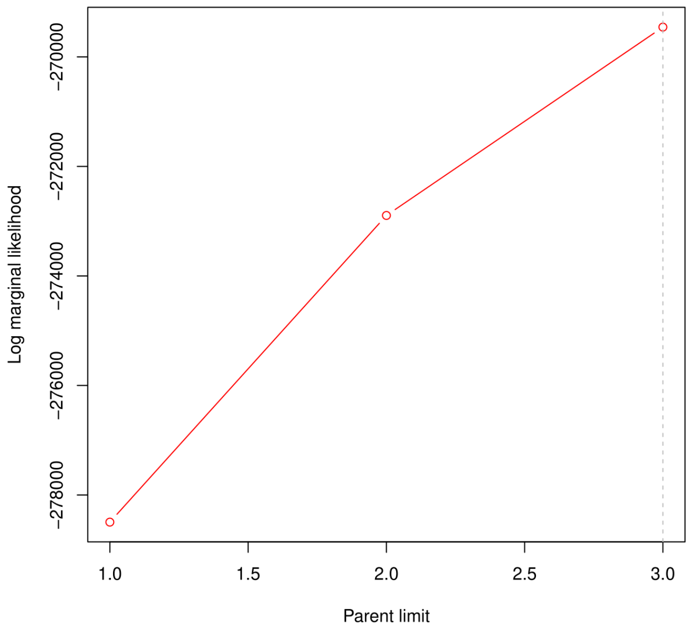

(B) HRS 2016-2022

Model fit for 1-3 parents/child

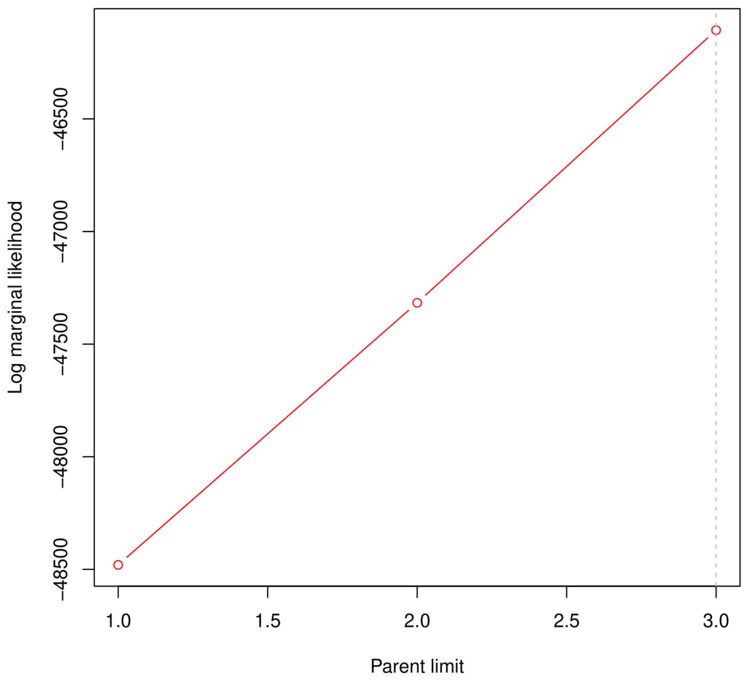

Figure legend:

Panel (A) shows model fit metrics for 1–3 parents per child in the **National Health and Nutrition Examination Survey (NHANES; 1999–2019)**, while Panel (B) presents corresponding results for the **Health and Retirement Study (HRS; 2016–2022)**. Model fit was evaluated using log marginal likelihood (logML) values to compare network complexity across configurations. The ABN framework was used to learn potential causal structures among biological aging markers and covariates under varying complexity constraints.

- **Abbreviations**:
  ABN – Additive Bayesian Network
  NHANES – National Health and Nutrition Examination Survey
  HRS – Health and Retirement Study
  logML – Log Marginal Likelihood
