# Supplementary material for: Telomere Length, Epigenetic Age Acceleration, and Mortality Risk in US Adult Populations: An Additive Bayesian Network Analysis
Source: Aging Cell. 2025 Jul 6;24(9):e70159. doi: 10.1111/acel.70159 (PMC12419851; doi:10.1111/acel.70159)
Supplement: Supplementary file 5 — Appendix S1. [file ACEL-24-e70159-s004.pdf]

SUPPLEMENTARY MATERIALS

APPENDIX I. DATABASES AND DETAILED STUDY DESIGN DOCUMENTATION

1) NHANES:

The CDC website on National Health and Nutrition Examination Survey (NHANES) provides comprehensive recommendations for researchers to interpret its data. This document encompasses survey methodology, sample design, estimating techniques, and analytical tactics. They are consistently revised to accommodate modifications in survey design and include novel statistical methodologies. The principal elements of the NHANES analytic standards encompass Plan and Operations Reports, Sample Design Documentation, Estimation and Weighting Procedures, and Analytic standards. The "National Health and Nutrition Examination Survey: Analytic Guidelines, 1999-2010" offers revised guidance for data analysis from those survey periods. The "NHANES Analytic Guidance and Brief Overview for the 2017-March 2020 Pre-pandemic Data Files" elucidates data collection difficulties encountered during the COVID-19 pandemic and offers directives for merging data from various cycles to achieve nationally representative estimates.

The present study used only demographics files from the 1999-2000 and 2001-2002 cycles, which were merged together and with other surplus sera data on epigenetic clocks and telomere length. While the demographics data covers all age ranges from 0 to 85y, the present study selected only those who were ≥50y of age to conform with the age range of DNAm data and epigenetic clocks. A two-cycle weighting procedure was carried out in most analyses using 4yr sampling weights adjusted to the epigenetic data availability.

Source: https://wwwn.cdc.gov/nchs/nhanes/analyticguidelines.aspx

2) HRS:

The Health and Retirement Study (HRS) offers detailed documentation on its survey design and methodology. The data collection path table provides a history of HRS data collection efforts, including links to detailed information about each data product. The longitudinal cohort sample design illustrates the accumulation of HRS samples over time, highlighting various birth cohorts. The study now follows a steady-state design, replenishing the sample every six years with younger cohorts. Tables provide detailed information on sample sizes and interview response rates for each survey year of the core biennial survey, categorized by overall panel, race/ethnicity, and cohort. Weight information is provided for unbiased national estimates, and resources such as "An Elementary Cookbook of Data Management using HRS Data with SPSS, SAS, and Stata Examples" provide practical guidance on managing and analyzing HRS data across different statistical software platforms. Technical reports offer in-depth descriptions of the HRS sample design, including methodologies like unfolding brackets to reduce item nonresponse in economic surveys. Imputations are discussed, detailing techniques applied to various survey waves. Administrative information provides insights into Institutional Review Board (IRB) considerations and other administrative aspects pertinent to the HRS. These resources collectively offer a thorough understanding of the HRS's survey design and methodology, supporting researchers in effectively utilizing the data for their analyses.

In addition to the Core data which can be linked to the tracker files, the RAND HRS data product can be downloaded at: https://hrsdata.isr.umich.edu/data-products/rand?_gl=1*65fx5r*_ga*MTA3MTg2OTA4NS4xNzMxNjIwNDk3*_ga_FF28MW3MW2*MTczNDE4OTM1MS41LjEuMTczNDE4OTM1Ni4wLjAuMA., with the latest version released in May of 2024 being used for this project (2020 HRS RAND FAT FILE, V1.A). The tracker file used for the current analysis is also the latest one that was recently released, released in November of 2024 and goes up to early 2022 in follow-up, with the URL: https://hrsdata.isr.umich.edu/data-products/cross-wave-tracker-file?_gl=1*1njsolk*_ga*MTA3MTg2OTA4NS4xNzMxNjIwNDk3*_ga_FF28MW3MW2*MTczNDE4O

TM1MS41LjEuMTczNDE5MDE0OS4wLjAuMA. The Health and Retirement Study (HRS) Tracker File is a comprehensive resource for researchers, providing a single record for each interviewee. It is updated with new information following each survey wave. The 2022 Tracker File includes data from all cohorts enrolled for the 2022 data collection. For our present study, it is mainly used to link HRS participants with date of death and thus estimating follow-up time in order to run various type of survival-type analyses.
Source: https://hrs.isr.umich.edu/documentation/survey-design

3) HANDLS

The Healthy Aging in Neighborhoods of Diversity across the Life Span (HANDLS) study aims to investigate the influences of race and socioeconomic status on age-related health disparities among African Americans and Whites in Baltimore, Maryland. The study employed several innovative recruitment strategies, including Mobile Research Vehicles (MRVs) for medical examinations and interviews, community engagement with local leaders and organizations, flexible scheduling for evenings and weekends, and regular follow-ups to maintain participant involvement. The HANDLS study faced several challenges, including mistrust of research due to historical abuses in medical research, logistical hurdles due to transportation barriers and safety concerns, and socioeconomic constraints for participants from lower SES backgrounds. To overcome these obstacles, the study engaged community leaders and ensured transparency in study objectives and procedures. Through these tailored strategies, HANDLS successfully recruited a cohort reflective of Baltimore's urban population, serving as a model for overcoming barriers in epidemiologic research involving urban populations.

The HANDLS study is a longitudinal epidemiological research initiative by the National Institute on Aging. It aims to investigate the influences of race and socioeconomic status (SES) on age-related health disparities, particularly concerning overall longevity, cardiovascular disease, and cerebrovascular disease. The study seeks to disentangle the relationship between race, SES, and health outcomes by addressing key questions:

- **Independent Influences**: What are the separate effects of race and SES on normal age-related functional changes and the incidence of age-related diseases?
- **Disease Progression**: How do these factors affect the natural history of common age-related diseases?
- **Contribution to Health Disparities**: In what ways do race and SES contribute to observed health disparities?
- **Early Biomarkers**: Are there early biomarkers of age-related health disparities that may enhance prevention or mitigation of disease severity?

To effectively address these questions, HANDLS employs mobile medical research vehicles (MRVs). These vehicles serve as community-based platforms for clinical research, facilitating the recruitment and retention of non-traditional research participants into age-related clinical studies. This innovative approach allows the study to reach populations directly within their neighborhoods, thereby enhancing participation rates and ensuring a more representative sample.

By integrating multidisciplinary assessments—including physical health evaluations, genetic analyses, and socio-demographic surveys—HANDLS aims to provide comprehensive insights into the complex interplay between race, SES, and health outcomes as individuals age. The ultimate goal is to inform strategies that can effectively reduce health disparities and promote healthy aging across various populations.

Sources: (Evans et al., 2010) and https://handls.nih.gov/

APPENDIX II. EPIGENETIC CLOCK DOCUMENATION

1) HEALTH AND RETIREMENT STUDY

A sample of 4,018 individuals from a subsample of HRS participants was used for data collection. High-quality DNA methylation data were achieved with over 97% sample success. DNA methylation was measured using the Illumina Infinium MethylationEPIC BeadChip.

Given commonality with NHANES and other studies, we opted to select five clocks, namely Horvath, Hannum, Levine PhenoAge, Grimm Age, and Dunedin Pace of Aging clocks. The first four were converted to an epigenetic age acceleration metric using the residual method using a linear model where chronological age was entered as the sole predictor. The residual can be interpreted as the number of years in biological age not accounted for by chronological age. These can be a fraction of a year or several years. Given that the Dunedin clock is already a pace of aging clock, no such modification was made. The five measures were then standardized z-scored after removing outliers within the final selected sample.
Source: https://hrsdata.isr.umich.edu/data-products/epigenetic-clocks and (Beydoun et al., 2022)

2) NHANES

Full documentation on DNA methylation data and epigenetic clocks is provided elsewhere: https://wwwn.cdc.gov/nchs/nhanes/dnam/. The documentation gives an overview of DNA methylation (DNAm) and epigenetic biomarker data from NHANES participants from 1999-2000 and 2001-2002,. Similar to HRS, the methodology used is the Illumina EPIC BeadChip arrays, with extensive bioinformatics preprocessing and normalization. The sample population is adults aged 50+ from various backgrounds. Quality control measures are in place to identify and remove outliers and mismatched samples. The data includes biomarker data, normalized DNAm matrices, and cell type proportions. Of the available clocks, many of which are used in HRS, we selected the most commonly analyzed clocks, namely Horvath, Hannum, PhenoAge, GrimAge, and Dunedin Pace of Aging clocks and analyzed them in a similar was as for HRS.

3) HANDLS

The HANDLS study used DNA methylation data from blood samples. Similar to HRS and NHANES, DNA methylation was measured using the Illumina Infinium MethylationEPIC BeadChip Illumina HumanMethylation EPIC array. Epigenetic age was computed using recognized clock methods like the Horvath clock amd Hannum clock. The anticipated epigenetic age was then compared to chronological age to determine epigenetic age acceleration (EAA), an indicator of the rate of biological aging relative to chronological age. DunedinPACE was used in HANDLS instead of DunedinPoAm.

Source: (Belsky et al., 2022; Beydoun et al., 2019; Beydoun et al., 2020; Evans et al., 2010)

APPENDIX III. TELOMERE LENGTH DOCUMENTATION

1) NHANES

The TELO_A and TELO_B data files from the NHANES 1999-2000 and 2001-2002 contain telomere length measurements for persons aged 20 and above. The telomere length assay utilized quantitative polymerase chain reaction (qPCR) to ascertain the telomere-to-single-copy gene ratio (T/S ratio), indicating telomere length in relation to a standard reference DNA. Each sample was analyzed thrice on three distinct days, yielding six data points per sample. Quality control measures used control DNA samples to standardize inter-run variability, with defined criteria for rejecting test runs and outliers to guarantee data precision. The intraassay coefficient of variation was 6.5%, signifying robust repeatability of the measurements. Researchers seeking to convert the T/S ratio to base pairs could utilize the following

formula: base pairs = 3,274 + 2,413 × (T/S). In the present study, NHANES data on telomeres was used focusing on mean telomere length (T/S). After excluding outliers, mean telomere length (TELO_MEAN) was converted to standardized z-scores within the final selected sample, and tertiles were also computed. *Sources*: https://wwwn.cdc.gov/Nchs/Data/Nhanes/Public/2001/DataFiles/TELO_A.htm and https://wwwn.cdc.gov/Nchs/Data/Nhanes/Public/2001/DataFiles/TELO_B.htm

2) HEALTH AND RETIREMENT STUDY

In 2008, the Health and Retirement Study (HRS) assessed telomere length in 5,808 participants aged 50 and above. Telome Health (now Telomere Diagnostics) performed the assay using qPCR, evaluating the telomere sequence copy number (T) in relation to a single-copy gene copy number (S), yielding a T/S ratio that correlates with average telomere length. Saliva samples were obtained via Oragene Collection Kits and DNA was extracted for examination. The interassay coefficient of variation was 6.5%, indicating robust repeatability of measurements.

Source: https://hrsdata.isr.umich.edu/data-products/2008-telomere-data

3) HANDLS

In the Healthy Aging in Neighborhoods of Diversity across the Life Span (HANDLS) study, telomere length was measured using qPCR. This method compares the amplification of telomeric DNA to a single-copy gene in a given sample. The ratio of telomeric DNA to single-copy gene DNA (T/S ratio) serves as a proxy for relative telomere length. DNA was extracted from blood samples, and the qPCR assays were conducted under controlled laboratory conditions to ensure accuracy and reproducibility. The T/S ratio was then used in analyses.
Source: (Cawthon, 2002; Evans et al., 2010)

APPENDIX IV. LASSO MODELS:

LASSO (Least Absolute Shrinkage and Selection Operator) is a regularization method used in regression to improve prediction accuracy and interpretability. It shrinks some coefficients to zero, effectively performing variable selection. There are three variants of LASSO: cross-validated LASSO (cvLASSO), adaptive LASSO, and minimum Bayesian Information Criterion (BIC) LASSO.

cvLASSO involves using cross-validation to determine the optimal penalty parameter (λ), which minimizes prediction error by splitting the dataset into training and validation subsets. The steps include standardizing predictor variables, performing k-fold cross-validation, and using the optimal λ for model estimation.

Adaptive LASSO assigns adaptive weights to the penalty term, enhancing variable selection consistency. The procedure involves fitting an initial regression model (e.g., OLS, logistic or Cox) and computing weights. The LASSO model with the weighted penalty term is estimated.

Minimum BIC LASSO selects the penalty parameter λ that minimizes the Bayesian Information Criterion (BIC). The procedure involves fitting LASSO models over a range of λ values and computing BIC for each model. This approach balances model complexity and goodness of fit.

Stata provides several key commands for implementing LASSO in regression models depending on the type of outcome (linear, logistic or cox). Only lasso linear was used in the present study to test predictors of telomere length across surveys of interest.

Source: https://www.stata.com/manuals/lasso.pdf

APPENDIX V. DISCRETE TIME HAZARD MODEL:

Discrete time hazard models are statistical methods used to analyze time-to-event data when the time variable is measured in discrete intervals, such as years, months, or days. These models are particularly useful in social sciences, public health, and educational research, where time is often measured in discrete units. Key features of discrete time hazard models include time disclosure, probability modeling, flexible covariates, and the binary logistic regression framework. The latter strength is a property that is useful for the implementation of additive Bayesian networks which can only accommodate gaussian, binomial and Poisson distributions for variables included in the model (See **Appendix VI**).

Steps in implementing discrete time hazard modeling include time period creation, baseline hazard specification, incorporating covariates, model estimation, and interpretation. Advantages of discrete time hazard models include effective handling of tied event times, incorporation of time-varying covariates, and simple implementation using logistic regression software.

Limitations of discrete time hazard models include discretization of continuous time data, loss of information when time intervals are large, and assumption of equal risk within each time interval. Applications include education, public health, and sociology. Common tools for implementing discrete time hazard models include R, Stata, and SAS.

Discrete time hazards models are logistic regression models applied to data in person-period format with several dummy variables included that would emulate a hazard function:

$$\log\left(\frac{h_t}{1-h_t}\right) = \beta_0 + \sum_{i=1}^{p} \beta_i X_i$$

Sources: (Kvamme & Borgan, 2021)

APPENDIX VI. ADDITIVE BAYESIAN NETWORKS:

A) Theoretical framework

Additive Bayesian networks (ABNs) are probabilistic graphical models that use a directed acyclic graph (DAG) to represent conditional interactions among variables. They offer benefits such as multivariate modeling, causal interpretation, and adaptability in managing various data types and distributions. ABNs are formulated by estimating local distributions for each node, employing linear regression for continuous data and logistic regression for binary variables. Bayes' Theorem is essential to ABN, as it aims to infer the posterior distribution of model parameters from the data. The software calculates the posterior distribution by integrating the probability obtained from the data with the designated priors. A scoring function is used to determine the ideal configuration for the ABN, and the Bayesian Information Criterion (BIC) is applied to balance goodness-of-fit and model complexity. ABN is widely used in disciplines like epidemiology, genetics, and social sciences to examine multivariate correlations and deduce causal pathways.

Sources: (Lewis & Ward, 2013; Scutari, 2022)

The following set of equations are used in this method:

(Eq. 1.1) *Linear regression*: $Y = \beta_0 + \sum_{i=1}^{k} \beta_i X_i + \varepsilon$

(Eq. 1.2) *Logistic regression*: $logit\left(P(Y = 1|X_1, \dots, X_k)\right) = \beta_0 + \sum_{i=1}^{k} \beta_i X_i$

(Eq. 1.3) *Likelihood Function*: $\boldsymbol{L(\theta|D) = \prod_{i=1}^{n} P(X_i \mid Parents(X_i), \theta_i)}$

(Eq. 1.4) *Bayesian Posterior*: $\boldsymbol{P(\theta|D) = \frac{P(D|\theta)P(\theta)}{P(D)}}$

(Eq. 3.5) *BIC for Model Selection*: $\boldsymbol{BIC = -2log\left(L(\theta|D)\right) + p \times log(n)}$

B) Additive Bayesian Network workflow

This appendix outlines the steps and R code used to perform additive Bayesian network (ABN) analysis, including data preparation, model specification, and iterative optimization.

Step 0: Install Necessary Packages

- Install R version 4.4 or higher.
- Install required R packages, including abn, INLA, BiocManager, graph, Rgraphviz, memisc, and others such as nnet, lme4, dplyr, and entropy.

Step 1: Data Preparation

1. **Load Data**:
    - Use the haven package to import a Stata .dta dataset.
    - Increase memory limits to handle large datasets if necessary.
2. **Data Wrangling**:
    - Convert selected variables (e.g., categorical ones) into factors.
    - Inspect the data structure using summary and structure functions.
3. **Define Variable Groups**:
    - Specify antecedent variables (e.g., AGE, SEX, NHB, etc.) and outcomes (d_var).

Step 2: Model Specification and Constraints

1. **Define Variable Distributions**:
    - Assign appropriate distributions to each variable, such as gaussian for continuous variables and binomial for categorical ones.
2. **Set Constraints**:
    - Create matrices to enforce banned and retained edges in the network:
        - **Banned edges**: Prevent arrows into antecedent variables and from final outcomes into other variables.
        - **Retained edges**: Ensure direct links between certain predictor variables and outcomes (e.g., dummy variables to d_var).
3. **Conflict Resolution**:
    - Resolve overlaps between banned and retained edges by prioritizing banned constraints.

Step 3: Optimization Across Parent Limits

1. **Iterative Analysis**:
    - Loop over different values of max.parents (e.g., 1 to 4) to optimize the network structure.

    - Save intermediate results for each parent limit and calculate the log marginal likelihood (mlik).
2. **Identify Optimal Parent Limit**:
    - Plot the relationship between parent limits and log marginal likelihood to determine the optimal value.
    - Save the network corresponding to the optimal parent limit.

Step 4: Build the Additive Bayesian Network

1. **Build Score Cache**:
    - Use the buildScoreCache function to precompute scores for potential directed acyclic graph (DAG) structures, adhering to defined constraints.
    - Limit the maximum number of parents (max.parents) for any node in the network to the optimal number chosen in Step 3 and based on coefficient strength.
2. **Search for Optimal DAG**:
    - Apply the searchHillClimber function to identify the optimal network structure using hill-climbing optimization.
3. **Fit the ABN Model**:
    - Use the fitAbn function to estimate network parameters and assess model performance.
    - Visualize the resulting DAG using the plotAbn function.

Output and Visualization

- Generate visual representations of the fitted DAG.
- Save the optimal network and associated data for future reference.
- Report log marginal likelihood scores and highlight the optimal network structure.

This code provides a comprehensive pipeline for conducting ABN analysis, including installation, data preprocessing, constraint specification, model fitting, and iterative optimization.

The optimal number of parents of child were determined in this study based on levelling off of the Log marginal likelihood and the desired level of complexity between key variables. Due to the heavy computational nature of this method and the relatively large sample (e.g. >18,000 person-period sample for NHANES 1999-2019), only up to 3 parents/child were considered. Thus, 2 parents/child were only considered if there was considerable levelling off of the marginal likelihood between 2 and 3 parents/child.

Source: https://r-bayesian-networks.org/

APPENDIX VII. GENERALIZED STRUCTURAL EQUATIONS MODELS

Generalized Structural Equation Modeling (*gsem*) in Stata is a flexible framework that estimates linear and non-linear relationships among variables, accommodating different dependent variable types and random effects. It expands the sem command and incorporates model-specific parameters for variable types and link functions. Unlike SEM, *gsem* relaxes assumptions, allowing for various distributions and their corresponding link functions. Models are estimated using Maximum Likelihood (ML) or Quasi-ML methods, including adaptive Gaussian quadrature when incorporating categorical latent variables or multiple levels. Goodness-of-Fit statistics and tests are available to evaluate model alignment with data. GSEM users can perform linear predictions, estimate residuals, and calculate linear and non-linear combinations of factors, particularly for assessing indirect effects. In this part of the analysis, the ABN-

selected DAG was recreated and evaluated, using both discrete time hazards specification of the final mortality outcome and parametric Weibull model with continuous time to event.

Probability density function for Weibull distribution

$$f(t;\delta,k) = \frac{k}{\delta}(\frac{t}{\delta})^{k-1}e^{-(\frac{t}{\delta})^{k}}, t \geq 0$$

Where:

- *t is the time or random variable of interst.*
- *δ>0 is the scale parameter*
- *k>0 is the shape parameter*

Cumulative distribution function (CDF) and survival function (complement of CDF) for Weibull distribution

$$F(t;\delta,k) = 1 - e^{-(\frac{t}{\delta})^{k}}, t \geq 0$$

$$S(t;\delta,k) = e^{-(\frac{t}{\delta})^{k}}, t \geq 0$$

Hazard function for Weibull distribution

$$h(t;\delta,k) = \frac{f(t;\delta,k)}{S(t;\delta,k)} = \frac{k}{\delta}\left(\frac{t}{\delta}\right)^{k-1}, t \geq 0$$

Source: https://www.stata.com/manuals/semgsem.pdf

SUPPLEMENTARY REFERENCES

Belsky, D. W., Caspi, A., Corcoran, D. L., Sugden, K., Poulton, R., Arseneault, L., . . . Moffitt, T. E. (2022). DunedinPACE, a DNA methylation biomarker of the pace of aging. *Elife, 11*. doi:10.7554/eLife.73420

Beydoun, M. A., Beydoun, H. A., Noren Hooten, N., Maldonado, A. I., Weiss, J., Evans, M. K., & Zonderman, A. B. (2022). Epigenetic clocks and their association with trajectories in perceived discrimination and depressive symptoms among US middle-aged and older adults. *Aging (Albany NY), 14*(13), 5311-5344. doi:10.18632/aging.204150

Beydoun, M. A., Hossain, S., Chitrala, K. N., Tajuddin, S. M., Beydoun, H. A., Evans, M. K., & Zonderman, A. B. (2019). Association between epigenetic age acceleration and depressive symptoms in a prospective cohort study of urban-dwelling adults. *J Affect Disord, 257*, 64-73. doi:10.1016/j.jad.2019.06.032

Beydoun, M. A., Shaked, D., Tajuddin, S. M., Weiss, J., Evans, M. K., & Zonderman, A. B. (2020). Accelerated epigenetic age and cognitive decline among urban-dwelling adults. *Neurology, 94*(6), e613-e625. doi:10.1212/WNL.0000000000008756

Cawthon, R. M. (2002). Telomere measurement by quantitative PCR. *Nucleic Acids Res, 30*(10), e47. doi:10.1093/nar/30.10.e47

Evans, M. K., Lepkowski, J. M., Powe, N. R., LaVeist, T., Kuczmarski, M. F., & Zonderman, A. B. (2010). Healthy aging in neighborhoods of diversity across the life span (HANDLS): overcoming barriers to implementing a longitudinal, epidemiologic, urban study of health, race, and socioeconomic status. *Ethn Dis, 20*(3), 267-275.

Kvamme, H., & Borgan, O. (2021). Continuous and discrete-time survival prediction with neural networks. *Lifetime Data Anal, 27*(4), 710-736. doi:10.1007/s10985-021-09532-6

Lewis, F. I., & Ward, M. P. (2013). Improving epidemiologic data analyses through multivariate regression modelling. *Emerg Themes Epidemiol, 10*(1), 4. doi:10.1186/1742-7622-10-4

Scutari, M., Denis, J.-B.,. (2022). *Bayesian Networks With Examples in R*. Boca Raton, FL: CRC Press.
